# Inspiratory Muscle Training in Patients with Heart Failure

**DOI:** 10.3390/jcm9061710

**Published:** 2020-06-02

**Authors:** Hugo Fernandez-Rubio, Ricardo Becerro-de-Bengoa-Vallejo, David Rodríguez-Sanz, César Calvo-Lobo, Davinia Vicente-Campos, J. L. Chicharro

**Affiliations:** 1 Facultad de Enfermería, Fisioterapia y Podología, Universidad Complutense de Madrid, 28040 Madrid, Spain; hugofern@ucm.es (H.F.-R.); ribebeva@ucm.es (R.B.-d.-B.-V.); davidrodriguezsanz@ucm.es (D.R.-S.); 2 Facultad de Ciencias de la Salud, Universidad Francisco de Vitoria, Pozuelo de Alarcón, 28223 Madrid, Spain; davinia.vicente@ufv.es; 3 Grupo FEBIO, Universidad Complutense de Madrid, 28040 Madrid, Spain; jlopezch@ucm.es

**Keywords:** heart failure, maximal respiratory pressures, resistance training, respiration

## Abstract

Background: Prior systematic reviews and meta-analysis addressed that inspiratory muscle training (IMT) improved inspiratory muscle weakness, cardiorespiratory fitness and quality of life similar to conventional exercise training as a first alternative in deconditioned patients with heart failure (HF) lead to a better adaptation to posterior exercise training. The heterogeneity and variability in a wide range of new studies about this topic led to the necessity of an updated and comprehensive narrative review. The present review aimed to analyze and update the most relevant studies about IMT in patients who suffer from HF. Methods: A narrative review was carried out about IMT in HF patients including 26 experimental studies divided into 21 clinical trials and 5 quasi-experimental studies identified through database searching in PubMed, Cochrane and PEDro. Results: There is enough evidence to state that IMT produces improvements in functional capacity of patients with HF. Nevertheless, there is not enough evidence to support that IMT could improve cardiovascular parameters, blood biomarkers or quality of life in these patients. Conclusions: Thus, IMT may be recommended to improve functional capacity in patients who suffer from HF; nevertheless, more evidence is needed regarding cardiovascular parameters, biomarkers and quality of life. Furthermore, mortality or HF hospitalization was not evaluated and most studies were not longer than 3 months. According to IMT protocols and study designs heterogeneity and mid-term follow-up, further investigations through high-quality long-term randomized clinical trials should be performed to achieve systematic reviews and meta-analysis to support strong evidence for IMT in HF patients.

## 1. Introduction

Worldwide, heart failure (HF) syndrome affects up to 23 million persons [1]. Furthermore, HF epidemic produces a key impact on quality of life, functional capacity and aging, as well as a high economic burden in the health system. HF may be considered as a multifactorial systemic disease involving structural, neuro-humoral, cellular and molecular mechanisms, which may be activated as a network in order to maintain physiological functioning. These complex and coordinated processes lead to an overload of the ventricles, an increased sympathetic-adrenal activity and a redistribution of the circulation, resulting in a complex clinical syndrome [2]. This syndrome may consequently produce an alteration of ventricular filling with or without reduction of the ejection fraction related to dyspnea, fatigue, exercise intolerance and peripheral and/or lung edema [1,3]. Indeed, HF syndrome is commonly divided into heart failure with reduced ejection fraction (HFrEF) and heart failure with preserved ejection fraction (HFpEF). Patients with an ejection fraction equal or greater than 50% were diagnosed with HFpEF, meanwhile patients with ejection fraction between 41% and 49% were alternatively diagnosed as HFrEF or HFpEF [1]. Patients with HF suffer from high morbidity and mortality rates, common hospitalizations and poor quality of life [4]. According to the New York Heart Association (NYHA) functional classification, HF severity may be classified as class-I without limitations or symptoms, class-II with slight limitations or symptoms during physical activity, class-III with important limitations to physical activity and class-IV with HF symptoms at rest, being exercise limitation considered as a main focus intervention in HF patients [5].

According to this consideration, exercise limitation may play a key role in HF due to an increased degree of exercise intolerance was associated with an unfavorable prognosis [6]. Patients who suffer from HF often experience an increased respiratory pattern and dyspnea during physical activity [6,7]. Despite the typical HF pathophysiological sequelae, there is not a clear relationship between cardiac function (i.e., left ventricular ejection fraction, left ventricular volumes and cardiac output) and exercise tolerance [6]. Indeed, hemodynamic abnormalities were initially considered as the main reason for these symptoms due to the ineffectiveness of the heart to increase cardiac output and pulmonary and systemic venous pressures. Increasing evidence supports a muscle hypothesis which suggested a deterioration of skeletal muscle as a source of HF symptoms [8]. Inspiratory muscle weakness in patients who suffer from HF seems to occur in a greater extent than lower limb musculature weakness [7,9,10]. This skeletal muscle atrophy may occur secondary to reduced cardiac output and tissue hypoxia, inflammation, increased systemic catabolism and prolonged immobilization, which may induce metabolic, structural, autonomic and functional changes in skeletal muscle [11]. These changes lead to protein degradation, increased levels of inflammatory cytokines (myokines), a change from slow-twitch (type-I) to fast-twitch (type-II) muscle fiber, a reduction in the number of mitochondria, impaired oxidative metabolism and early acidosis. Consequently, a reduction in muscle resistance, activation of afferent reflexes (meta-reflex) and a sustained increase in sympathetic-adrenal activity are presented. In addition, ventilation alterations increase fatigue and dyspnea as well as decrease aerobic capacity [11,12]. Thus, HF has been commonly associated with inspiratory muscle weakness, and the reduction of this inspiratory muscle weakness could have the potential to improve many secondary HF effects [7,13]. Inspiratory muscle weakness was associated with an increase in muscle meta-reflexes, which may play a key role in the clinical status of patients who suffer from HF [14].

Regarding muscle meta-reflex, this reflex may be considered as a blood pressure regulator, cardiac output, and regional distribution of muscle blood flow, involving chemically sensitive receptors located in the muscle parenchyma, which are activated by metabolites during muscle contraction [15,16]. Muscle afferent fibers of the meta-reflex are mainly comprised by unmyelinated group-IV neurons, whose receptors are chemically sensitive to metabolites produced by skeletal muscle contraction [15,16,17]. The specific type of metabolites that may activate the meta-reflex remains controversial. Some specific metabolites such as lactic acid, potassium, adenosine, arachidonic acid, diprotonated phosphate, prostaglandins or hydrogen ions have been proposed in order to activate this meta-reflex [17,18]. The efferent response secondary to meta-reflex activation may be an increased sympathetic nerve activity that could constrict the systemic vessels and increase blood flow in the contracted or active muscle, while evoking cardiac ionotropic and chronotropic effects to increase cardiac output. Therefore, this meta-reflex provokes a sympathetic-adrenal response that raises blood pressure during exercise and allows redistribution of muscle blood flow and volume, including respiratory muscles [15].

Thus, this meta-reflex may occur in respiratory muscles as a result of fatigue during an effort that may lead to metabolic sub-products accumulation, triggering the activation of receptors and consequently the meta-reflex of these muscles [15,16,18,19,20,21]. This meta-reflex activates the sympathetic nervous system response, generating a peripheral vasoconstriction, a decrease in blood flow of skeletal muscle perfusion, an increase of exercise-induced fatigue and a redistribution of blood flow to the respiratory muscles in order to maintain their function [15,19,21,22,23]. This increase in skeletal muscle fatigue may lead to decreased exercise tolerance and muscle strength development [19,21]. Furthermore, respiratory muscles meta-reflex can lead to an increase of heart rate and blood pressure as well as a reduction of blood flow in the renal and mesenteric arterial vessels. Simultaneously, these respiratory muscles can be influenced by both meta-reflex and chemo-reflex [18].

In addition to the meta-reflex, chemo-reflex may be considered as one of the main mechanisms to control the ventilatory and autonomic response secondary to changes in arterial oxygen, carbon dioxide and pH concentrations. Indeed, central chemoreceptors, which are located on the ventral surface of the medulla (medulla oblongata), seem to respond primarily to variations in CO_2_ partial pressure (PCO_2_) in arterial blood flow. Peripheral chemoreceptors, through type-1 glomus cells which are located in the common carotid artery and in the aorta artery, with afferences to the respiratory center which are also located in the bulb and in the solitary tract nucleus, mainly seem to respond under variations in the O_2_ partial pressure (PO_2_) in arterial blood flow [18,22]. An increase in the sympathetic nervous system activity may be secondary caused to central or peripheral chemo-reflex. Consequently, heart rate and blood pressure increases are produced, contributing to the development of hyperventilation during exercise carried out by patients with HF, showing an increase of the respiratory muscles’ activity [6,22]. Meta-reflex may be considered as a powerful activator of the central chemo-reflex. According to Ribeiro et al. [22], inspiratory musculature weakness increases the peripheral chemo-reflex. Thus, this weakness of respiratory muscles may excessively increase the sensitivity to chemo-reflex by both central and peripheral responses in patients with HF, causing peripheral chemo-reflex activation at a lower threshold. This condition may lead to a sustained increase in sympathetic nervous system activity, allowing secondary adrenergic vasoconstriction and increases in right and left ventricular afterloads, being sympathetic hyper-activation considered as a key predictor of HF mortality. Therefore, inspiratory muscle strength reduction may increase the chemo-reflex and meta-reflex sensitivity in patients who suffer from HF, which could be related to reduced functional capacity and exercise intolerance [22,24]. Thus, inspiratory muscle strength may modulate meta-reflex [15,16,18,19,20,21] and chemo-reflex [18,22], which could lead to modify respiratory system alterations and systemic symptoms like fatigue, functional capacity or quality of life [22,24].

Indeed, respiratory system alteration may be considered as one of the main factors that limit exercise capacity in patients who suffer from HF, secondary to impaired perfusion and/or ventilation [6,25,26,27,28]. Factors which limit perfusion under HF condition may include poor right ventricular performance, elevated pulmonary arterial pressure and high pulmonary vascular resistance [25,28,29]. Indeed, inspiratory musculature weakness may be considered as one of the key factors that can limit ventilation in patients who suffer from HF [26,27]. Inspiratory muscle weakness is presented in 50% of HF patients [30] and contributes to a poor prognosis in these patients [22]. This condition is diagnosed if the maximum inspiratory pressure (PImax) is lower than 70% compared to the normalized values according to patients’ age and sex [7,30] or PImax is lower or equal to 60 cmH2O [31]. PImax may be related to the dyspnea perception during daily activities and serve as a prognostic indicator in patients who suffer from HF [23,24,32,33]. Fatigue and dyspnea symptoms usually suffered by patients with HF may be partially secondary to respiratory muscles weakness [7,21,24,27,34,35,36], due to respiratory muscle strength reduction, which could require a greater PImax fraction during respiration. Therefore, patients may experience greater dyspnea intensity secondary to increase of PImax fraction used during respiration [37]. This weakness of the respiratory musculature is also associated with a decrease in tidal volume, which may increase the ventilation-dead space ratio, increasing the ventilation-perfusion mismatch during exercise in patients who suffer from HF. In addition, an increase in the correlation between ventilation and CO_2_ is generated as a key prognostic indicator in patients who suffer from HF [21,27,36,37]. Finally, the inspiratory muscles weakness promotes their fatigue, leading to an early activation of the metabolon-receptors in these muscles with possible histological changes of respiratory muscle fibers [22].

Thus, respiratory muscle weakness is accompanied by histological changes. Indeed, biopsies of the respiratory muscles performed in patients with HF have shown a lower percentage of type-IIx and type-IIa muscle fibers and a higher percentage of type-I muscle fibers, compared to healthy individuals [11,38,39,40], being these modifications different from those observed in the limbs skeletal muscles [11]. Although the proportion of type-I fibers is usually increased in the respiratory muscles, an atrophy of these fibers has been also found under HF [21,30,40]. Furthermore, a higher percentage of type-I fibers is related to a greater oxidative enzymatic activity in these patients. These changes may be probably induced by myogenic regulatory factors linked to the increased sustained effort of ventilation. This adaptation facilitates an increase in respiratory endurance, with a parallel decrease in the maximum strength and muscular power of these muscles, leading to other compensatory mechanisms to maintain respiratory function [24,38].

With regard to other mechanisms that influence respiratory function in addition to the inspiratory muscle weakness of patients with HF, their respiratory activity is increased due to respiratory muscles having to work against increased resistive and elastic loads in these patients [6]. Elastic load is increased as a consequence of increased pulmonary tissue stiffness due to competition between the lung and heart tissue for the intrathoracic space (i.e. cardiomegaly), congested pulmonary and/or bronchial vascular flow as well as pulmonary interstitial edema. This increased resistive load may lead to lung congestion with a limitation of expiratory flow and sustained hyperventilation [6,7,21], which may result in increased oxygen and blood flow requirements of the active respiratory muscles. Given that HF may be often accompanied by a limitation in the response of cardiac output to exercise, the appearance of fatigue may be produced in an early way [6]. Furthermore, HF patients present a maladaptive respiratory pattern with a shorter expiratory time and a longer inspiratory time due to inspiratory muscles weakness and reduced inspiratory resistance [41]. This condition may also cause sympathetic-adrenal hyperactivity, being this condition a predictor of mortality under HF [22], because parasympathetic activity in the sinus node is decreased during inspiration and parasympathetic activity is upregulated during expiration, which hinders the functional capacity of these patients [42].

In this regard, Yamada et al. [21] demonstrated in patients who suffered from HF the relationship between respiratory muscles weakness and functional capacity limitation during a 6-minute walking test, regardless of suffering from a restrictive respiratory pattern or lower limb skeletal muscle weakness. Furthermore, inspiratory muscle weakness was related to different functional classes of NYHA, showing the highest inspiratory force for patients with class-I condition and the lowest inspiratory force for patients with class-IV, inspiratory muscle training (IMT) being considered as a possible key rehabilitation intervention to improve this symptomatology in patients suffering from HF [24,41].

Cardiac rehabilitation is a well-studied and comprehensive rehabilitation program that has been proven to improve functional capacity in heart failure patients. Nevertheless, these cardiac rehabilitation programs are currently underutilized [43]. IMT may serve as a useful alternative that may be more amenable to HF patients’ participation. Furthermore, IMT may serve populations excluded from cardiac rehabilitation, such as patients unable to perform exercise, being an interesting treatment option for clinicians [33,44].

According to these antecedents, prior systematic reviews and meta-analysis have addressed that IMT improved inspiratory muscle weakness, cardiorespiratory fitness and quality of life similarly to conventional exercise training as a first alternative in deconditioned patients with HF, leading to a better adaptation to posterior exercise training [33,44]. In 2013, Smart et al. [44] analyzed 11 controlled trials of IMT in chronic HF patients including data on 287 patients divided into 148 patients who received IMT and 139 patients who were assigned to sham or sedentary control groups. In 2014, Montemezzo et al. [33] analyzed 9 randomized controlled clinical trials including 240 patients and comparing IMT with controls or sham interventions. To date, there is a lack of literature reviews about IMT in patients with HF and several experimental studies, including clinical trials, quasi-experimental studies and clinical cases, have been published. The heterogeneity and variability on a wide range of new studies about this topic led to the necessity of an updated and comprehensive narrative review. The present review aimed to analyze and update the most relevant studies about IMT in patients who suffer from HF.

## 2. Methods

### 2.1. Study Design

A narrative review was carried out following the applicable recommendations of the Preferred Reporting Items for Systematic Review and Meta-Analyses (PRISMA) criteria [45]. This narrative review was performed in order to update the available data from prior systematic reviews about IMT in patients who suffer from HF [33,44] but including different studies types, such as clinical trials, quasi-experimental studies and case-series, published up to January 2020. 

### 2.2. Search Strategy

Database searching process was carried out during January 2020. PubMed, Cochrane and PEDro were the used databases for this process. Restrictions used in this database search were experimental studies carried out only in humans with access to full texts using the following search strategy (“Inspiratory muscle training” OR “respiratory muscle training”) AND “heart failure” in the title of abstract from studies which were written in Spanish or English languages.

### 2.3. Selection Criteria

Inclusion criteria comprised experimental studies, such as clinical trials, quasi-experimental studies or clinical cases, including HF who received IMT intervention. Exclusion criteria comprised non-experimental studies as well as systematic reviews or meta-analyses and studies which included patients with other conditions in addition to HF such as strokes, lung hypertension, “Fontan circulation” or mitral valve alterations, among other pathologies. All possible outcome measurements were accepted for review because our aim was to update all information and assess variable experimental studies in HF patients who were treated with IMT in an isolated form or in combination with other interventions [33,44].

### 2.4. Data Extraction

Studies characteristics such as sample size, socio-demographic data and baseline measurements as well as training protocol, including duration, frequency, intensity, IMT device and protocol were registered. These data were divided into 3 tables including characteristics of the randomized clinical trials without other interventions (Table 1a), characteristics of the randomized clinical trials with other interventions (Table 1b) and characteristics of the quasi-experimental studies and clinical cases (Table 1c).

Data extraction comprised study citation, group and sample sizes, outcome measurements with pre- and post-intervention means ± standard deviations (SD), statistical significance (*p*-values), and additional information about outcome measurements procedure was provided when it was necessary. These data extraction was divided into several tables including inspiratory muscle strength and resistance (Table 2a), lung function (Table 2b), dyspnea (Table 3a), fatigue (Table 3b), functional class and capacity (Table 4), strength in limbs (Table 4b), parameter related to VO_2_ (Table 5), VE/VCO_2_ and VE (Table 6), cardiovascular parameters (Table 7), biomarkers (Table 8) and quality of life (Table 9), according to the updated literature and prior reviews [33,44].

## 3. Results and Discussion

### 3.1. Flow Diagram

**Figure 1:**
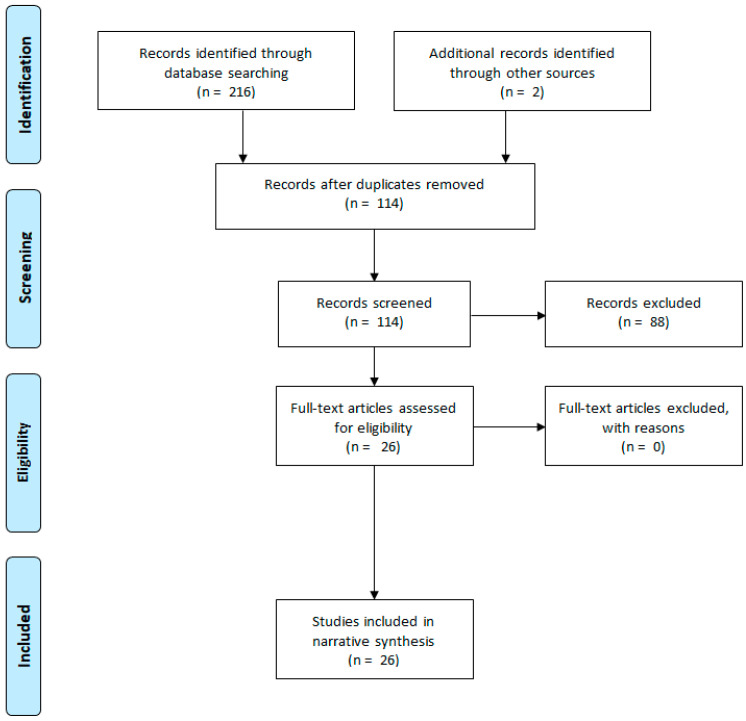
Flow diagram of the narrative review.

From 218 records identified through the searching process, 192 records were removed due to duplicates and exclusions (Supplemental Table S1), and finally, 26 studies were included in narrative synthesis (Figure 1).

### 3.2. Inspiratory Muscle Training

Inspiratory muscle weakness observed in patients with HF is reversible. Thus, inspiratory muscle training (IMT) is one of the key interventions for the improvement of the strength and inspiratory muscular resistance in patients who suffer from HF [46].

IMT may be considered as a training method by workloads applied during inspiration [33]. IMT can be performed in three different ways by an inspiratory load threshold device, a resistive load threshold device and an isocapnic hyperpnea. First, IMT using inspiratory load threshold devices is applied using an inspiratory pressure to cause the valve openness and thus allow air flow to pass during inspiration. Second, IMT using resistive loading devices is applied by several holes of different diameter that provide resistance to inspiration using a smaller diameter of the hole to provide a greater resistance. Third, IMT using an isocapnic hyperpnea device can be only performed in a well-equipped respiratory physiology laboratory and is applied maintaining a certain level of ventilation in the form of volitional hyperpnea for 12 min mean while CO_2_ is added to the inspired air in order to maintain isocapnia in arterial blood flow [47].

To date, 26 articles have examined the effects of IMT in patients with HF (Table 1) [20,30,31,35,48,49,50,51,52,53,54,55,56,57,58,59,60,61,62,63,64,65,66,67,68,69]. Twenty-one of these studies were randomized clinical trials [20,30,31,35,49,50,51,52,53,54,55,56,57,59,60,61,62,63,66,67,68,69] (Table 1a,b), four studies were quasi-experimental studies [48,58,64,65], and one study was a clinical case [59] (Table 1c). The sample size for most of these studies was small. Most of the studies were carried out in patients with HFrEF [20,30,31,35,48,49,50,51,52,53,54,55,59,60,61,62,63,64,65,66,67,68,69]. In addition, patients of the study carried out by Cahalin et al. [65] were waiting for a heart transplant. Furthermore, three studies applied IMT to patients with HFpEF [56,57,58]. IMT modality used in most studies was inspiratory load threshold training [30,31,35,48,49,50,51,54,55,56,57,58,60,61,63,65,66], and eight studies used inspiration resistance training [52,53,59,62,67,68,69]. The study carried out by Moreno et al. [20] gave to the IMT group the option to use a threshold load device or a resistive load device. Finally, the study performed by Mancini et al. [64] used the inspiratory load threshold training and isocapnic hyperpnea.

From the total sample of clinical trials [20,30,31,35,49,50,51,52,53,54,55,56,57,59,60,61,62,63,66,67,68,69], 14 randomized clinical trials without other interventions compared IMT versus control or sham interventions, and their citations, sample characteristics and training protocol explanation were detailed in Table 1a.

Regarding the total sample of clinical trials [20,30,31,35,49,50,51,52,53,54,55,56,57,59,60,61,62,63,66,67,68,69], 7 randomized clinical trials with other interventions showed that most studies were compared or combined with physical training programs including 6 studies which added physical training program for both control and IMT groups as well as 3 studies whose IMT arm only received physical training program. Citations, sample characteristics and training protocol explanation for these studies were detailed in Table 1b.

Other experimental studies included 4 quasi-experimental studies [48,58,64,65] and one clinical case [59]. Two of the quasi-experimental studies compared IMT with control or healthy groups. In addition, citations, sample characteristics and training protocol explanation for these experimental studies were detailed in Table 1c.

In most of all these studies, the intervention group only performed IMT, and the control group used a simulated IMT (no load), a low intensity fixed load or only received education [20,30,31,35,48,49,51,54,55,56,58,63,65,66,67,68,69]. The remaining 9 studies also included a physical training program in addition to performing IMT in the intervention group [50,52,53,57,59,60,61,62,64]. IMT programs were mainly differentiated into four variables, such as the percentage of the PImax or the sustained maximum inspiratory pressure (SMIP), the duration of the training session, the weekly frequency of IMT and the total duration of the training program. PImax percentages varied from 20% to 60% in the intervention groups, with 30% being the most used PImax percentage. The SMIP ranged from 15% to 60%. Total training periods ranged from 4 to 12 weeks; durations for each IMT session varied from 15 to 30 min, and frequencies varied from two daily sessions to three weekly sessions.

#### 3.2.1. IMT Effects on Respiratory Muscle Performance and Lung Function 

Most studies measured PImax, showing a significant increase (*p* < 0.05) of this parameter for the group which received IMT [20,30,31,35,48,49,50,51,52,53,54,55,56,58,59,60,62,63,64,65,66,67,68,69]. Nevertheless, the comparison of both IMT and control groups did not reveal statistically significant differences (*p* > 0.05) for this parameter in 4 studies [52,53,68,69]. Only 2 studies did not measure PImax [57,61]. Maximum expiratory pressure was evaluated in 7 studies [35,50,51,59,60,64,65]. All these studies showed a significant improvement, except for the study carried out by Weiner et al. [35]. Inspiratory muscle endurance (IME) was measured in 5 studies and observed a significant increase [30,35,50,54,64]. Weiner et al. [35] also observed an improvement in expiratory muscle endurance (EME) showing statistically significant differences. A secondary measure of IME (SMIP) was examined in 6 studies [52,53,63,67,68,69] and also increased significantly. Indeed, both PImax and IME showed significant improvements from the first week of IMT [20,54] (Table 2a).

Lung function was measured in nine studies [30,35,51,53,59,60,64,67,68] and demonstrated that IMT did not seem to be a useful intervention to improve significantly pulmonary function because statistically significant differences (*p* < 0.05) for some parameters were only shown in 3 studies [35,51,68]. Forced vital capacity (FVC), forced expiratory volume in the first second (FEV1), FEV1/FVC ratio, maximum expiratory flow and vital capacity were the parameters used to determine lung function. FVC increased statistically significant in 2 studies [35,68]. FEV1 showed statistically significant differences in only 1 study carried out for Laoutaris et al. [68]. FEV1/FVC ratio and maximum expiratory flow were statistically and significantly increased in the study performed by Bosnak-Guclu et al. [51]. Vital capacity did not show statistically significant differences (*p* > 0.05) regarding studies that assessed this parameter [59,64]. Training intensity equal or greater than 40% showed statistically significant improvements for one or more lung function parameters in 3 studies [35,51,68] from the total of 5 studies [35,51,53,67,68] that used this training intensity (Table 2b).

Indeed, other important data to highlight are provided in the following 3 studies. Mancini et al. [64] observed an improvement (*p* < 0.05) in maximum voluntary ventilation without variation in the ti/ttot ratio. Chiappa et al. [48] showed an increase in the thickness of the diaphragm in addition to a hypertrophy of the diaphragm that was associated with a statistically significant increase (*p* < 0.001) in PImax. Finally, Laoutaris et al. [67] reported an increase in the inspiratory volume (*p* < 0.001).

In summary, IMT may be considered as a useful therapy to improve the strength and endurance of the respiratory muscles. Authors encouraged researchers to assess lung function after performing IMT in future studies dividing the groups into one that performed IMT at an intensity between 20% and 30% of the PImax or SMIP and the other group at an intensity of ≥40% of the PImax or SMIP in order to observe if higher intensity improvements in lung function may be achieved.

#### 3.2.2. Dyspnea

IMT effects on dyspnea were measured in 15 studies [30,31,35,51,52,53,54,61,63,64,65,66,67,68,69] and demonstrated that IMT may be a useful treatment to reduce dyspnea at both rest and during exercise performance. All these studies showed a significant improvement (*p* < 0.05) in dyspnea except for the study carried out by Johnson et al. [66]. Most studies used the Borg scale to assess dyspnea [30,31,52,53,64,65,66,67,68,69], and only 2 of them did not observe a statistically significant difference (*p* > 0.05) in the dyspnea sensation [64,66]. Nevertheless, Mancini et al. [64] did not observe statistically significant improvements for dyspnea; patients walked a greater distance with the same dyspnea sensation and concluded that HF patients were able to increase working charge without dyspnea sensation increase. Three studies used the Modified Medical Research Council (MMRC) scale [51,54,61] showing statistically significant improvements with respect to the control group, except for the study carried out by Marco et al. [54], which did not show statistical significance. Two additional studies measured the dyspnea index [35] and the Mahler transition index [63]. Weiner et al. [35] revealed statistically significant differences. Nevertheless, Martínez et al. [63] did not reported these improvements. Laoutaris et al. [68] correlated as statistically significant (*p* < 0.01) the decrease in dyspnea with an increase in the distance covered in the 6 min walking test (Table 3a).

Fatigue was only analyzed in 2 studies, and both studies showed a fatigue decrease after IMT intervention [51,61], although only the study carried out by Hossein Pour et al. [61] showed statistically significant differences (*p* < 0.05) with respect to the control group. Both studies used the Fatigue Severity Scale (FSS) to assess fatigue [51,61] (Table 3b).

Thus, IMT may reduce the sensation of dyspnea in these patients, both at rest and during exercise performance. Authors encourage researchers to carry out further studies to obtain more evidence regarding the decrease in fatigue after the use of IMT.

#### 3.2.3. Exercise and Functional Capacity

Exercise functional capacity was assessed in 18 studies [30,35,50,51,52,53,54,56,57,59,60,61,62,63,64,66,67,68] using different parameters. Most studies used gait tests to assess functional capacity, and 10 studies [30,50,51,56,57,60,63,64,67,68] used the 6-minute walking test showing that IMT group produced a significant improvement (*p* < 0.05) in the distance compared to the control group, except for 3 of them [50,57,66]. Weiner et al. [35] used the 12-minute walking test, and the study carried out by Johnson et al. [66] used the “corridor walk test” (CWT). Weiner et al. [35] demonstrated statistically significant improvements, while Johnson et al. [66] did not report statistically significant differences (*p* > 0.05). Two of the studies presented both control and IMT groups performing a general physical training program and did not show statistically significant differences between groups [50,57]. The improvement in the 6-minute walk test was related to the improvement in PImax (*p* < 0.01) and SMIP (*p* < 0.05) [63] (Table 4a).

Eight studies evaluated the time to exhaustion at a given exercise intensity [30,52,53,64,66,67,68,69]. In all these studies, an improvement was shown in favor of the IMT group (Table 4a), except for 3 studies [53,66,69]. Quadriceps strength was measured in 5 studies [51,52,59,60,62], showing statistically significant improvements (*p* < 0.05) in favor of the IMT group for all reported studies. In these research reports, three studies [52,60,62] showed that IMT group also performed strength training, and the control group did not carry out this training or trained at a lower intensity. Strength for upper limbs was examined in three studies [54,59,60], and significant improvements in favor of the IMT group were only shown in the study carried out by Kawauchi et al. [60], which used a control group with strength training at a lower intensity than in the IMT group. Furthermore, Marco et al. [54] did not find a statistically significant correlation (*p* > 0.05) between the improvement of PImax or PEmax and the grip strength (Table 4b).

The study carried out by Bosnak et al. [51] was the only study that evaluated and observed a significant improvement (*p* < 0.05) in balance in the IMT group. This fact may be probably due to the increase in the diaphragm thickness after performing a relatively short IMT period (4 weeks) [48]. Thus, greater strength and resistance of the inspiratory muscles may contribute to better balance, since the diaphragm and other breathing muscles may be responsible for maintaining both breathing and balance [27,48,51,52,55]. Therefore, peripheral and central adaptations of IMT may improve the mechanisms responsible for respiration, ventilation and balance and subsequently produce a synergy between these processes [27].

Four studies [52,53,60,61] assessed functional capacity by the NYHA scale. Form these 4 studies, the study carried out by Hossein Pour et al. [61] was the only research that observed between-groups statistically significant differences (*p* < 0.05), although the other 3 studies [52,53,60] showed an IMT intra-group improvement but did not show statistically significant differences (*p* > 0.05) with respect to the control group. These results may be due to isolated IMT improved NYHA functional class, although IMT in conjunction with physical exercise did not support additional benefits (Table 4a).

In conclusion, IMT may improve the distance covered in walking tests and endurance to maintain a certain exercise. Nevertheless, higher quality studies are needed using isolated IMT in one group to demonstrate significant improvements in limb strength or functional NYHA class. Regarding endurance to maintain a certain exercise level until exhaustion, current evidence is ambiguous to assess the effect of IMT on limb-strength increase. Higher quality studies would be necessary considering the following two aspects. Firstly, examining whether isolated IMT could improve this parameter since only two studies [51,54] used isolated IMT. Secondly, observing if IMT could provide additional benefits to certain training methods assessing if both IMT and control groups could obtain differences using a different IMT protocol and the same training protocol.

#### 3.2.4. Metaboreflex Activity

Three studies [20,48,55] analyzed the IMT effects on the meta-reflex of respiratory muscles, demonstrating a statistically significant improvement (*p* < 0.05) in blood flow in the limb muscles at rest or during exercise due to a decrease in limb vascular resistance. IMT significantly increased the ventilatory load required to provoke the peripheral vasoconstriction mediated by respiratory muscles meta-reflex. This fact may be due to a greater resistance to fatigue in these muscles, reducing the accumulation of metabolites that triggered the meta-reflex activation [19,20,48,55,70]. Thus, IMT may be associated with an effect of inspiratory muscle release by increasing the inspiratory muscles thickness, strength and diaphragmatic muscular aerobic capacity. This effect was associated with less accumulation of metabolites and reduced peripheral vasoconstriction, resulting in increased peripheral blood flow and greater exercise tolerance [19,27,48]. According to this decrease in meta-reflex, Mello et al. [55] observed that IMT reduced muscle sympathetic activity improving sympathetic and vagal modulation of the cardiovascular system in patients with HF. Furthermore, this decrease in sympathetic-adrenal activity could be also due to a reduction in chemo-reflex since IMT could improve oxygen saturation. In addition, Moreno et al. [20] showed that respiratory muscles meta-reflex decreased after IMT. Thus, an oxygen saturation improvement of the intercostal and forearm muscles was observed. 

Therefore, IMT may reduce inspiratory muscles’ meta-reflex in patients who suffer from HF.

#### 3.2.5. Peak VO_2_

Sixteen studies evaluated peak VO_2_ [30,35,50,52,53,55,56,57,58,59,62,63,64,67,68,69] and showed significant improvement (*p* < 0.05) in the IMT group, except in the study carried out by Weiner et al. [35] which showed an improvement in only 3 out of 10 patients. Six studies [35,52,69] did not show significant differences between groups (*p* > 0.05). Two studies [30,68] increased peak VO_2_, which was correlated with the improvement of PImax and SMIP. Nevertheless, three studies [58,69,71] did not find this correlation. In addition, inverse and statistically significant correlations were shown between the dyspnea percentage decrease and peak VO_2_ increase (*p* < 0.05) [69], as well as VO_2_ improvement and baseline PImax percentage (*p* = 0.04) [50] (Table 5).

In addition to peak VO_2_, three parameters related to peak VO_2_ were measured, such as anaerobic threshold VO_2_, circulatory power (CP) and oxygen absorption slope (OUES). Five studies [56,64,67,68,69] analyzed VO_2_ value at the anaerobic threshold, showing a statistically significant improvement (*p* < 0.05) only in the study performed by Palau et al. [56]. Three of the studies [67,68,69], which did not show this improvement (*p* > 0.05), determined a significant VO_2_ value decrease at the anaerobic threshold in the control group while the IMT group maintained these values.

The circulatory power was calculated as the product of the VO_2_ peak by the maximum systolic blood pressure [30,50,52,53,69]. Two of these studies [53,69] did not show statistically significant differences (*p* > 0.05). Oxygen absorption efficiency slope (OUES) values were analyzed in two studies [49,50] showing a statistically significant improvement (*p* < 0.05) of these values in the IMT group. In addition, Laoutaris et al. [69] observed a significant improvement in oxygen pulse (VO_2_/HR) in the IMT group, but there were not statistically significant differences with respect to the control group.

In conclusion, IMT may increase peak VO_2_ and circulatory power. Regarding OUES, CP and oxygen pulse, IMT could improve these parameters, although a larger number of studies should be carried out. Finally, IMT did not seem to be a useful therapy to improve VO_2_ anaerobic threshold.

#### 3.2.6. VE/VCO_2_

Eight studies [30,50,52,53,64,67,68,69] analyzed IMT changes produced on ventilation per minute (VE). All these studies showed a significant intragroup improvement (*p* < 0.05) in favor of the IMT group, except for 3 studies (*p* > 0.05) [52,68,69]. Only 5 studies [30,50,64,67] revealed statistically significant differences for the IMT group compared to the control group (Table 6).

Fourteen studies [30,50,52,53,55,56,57,59,62,63,64,67,68,69] measured the IMT effects on the VE/VCO_2_ slope showing controversial findings because seven studies [30,50,55,56,57,59,68] demonstrated a significant effect (*p* < 0.05) in favor of the IMT group, while the remaining seven studies did not observe statistically significant differences (*p* > 0.05) between IMT and control groups [52,53,58,62,63,67,69]. Winkelmann et al. [50] showed a significant negative correlation (*p* < 0.01) between the improvement in PImax and the decrease in the VE/VCO_2_ slope.

Therefore, there is a lack of evidence in order to affirm that IMT may reduce the VE/VCO_2_ slope.

#### 3.2.7. Cardiovascular Parameters

Different cardiovascular parameters were evaluated after IMT, such as heart rate (HR), left ventricular ejection fraction (LVEF), left ventricular diameter at the end of systole/diastole, left atrial volume index, heart rate variability and blood pressure [20,50,51,52,53,55,56,57,67,68,69]. Nine studies [20,50,52,53,55,56,67,68,69] measured changes in maximum or resting HR, although the findings of these studies showed a high variability of results (Table 7).

Four studies assessed LVEF after performing IMT and did not show significant differences (*p* > 0.05) between groups [52,53,56,67], although intra-group statistical significance (*p* < 0.05) was shown in two studies [52,53]. The left ventricular diameter at the end of systole or diastole was also evaluated [52,53,56,67]. Only two studies carried out by Laoutaris et al. [52] and Adamopoulos et al. [53] showed an intragroup significant decrease in left ventricular diameter at the end of diastole observed in both IMT and control groups, being the only two studies that combined IMT with strength training. Thus, these improvements may be due to the training program. Finally, the left atrial volume index was measured in two studies [56,57], showing a significant improvement in the IMT group after 6 months according to Palau at al [57].

Heart rate variability was assessed in two studies [55,69], determining a significant increase (*p* < 0.05) in this parameter in the IMT group according to Mello et al. [55]. Laoutaris et al. [69] justified the absence of improvement because the evaluation was carried out for 24 h, and the patients in that period carried out physical exercise. This fact supposed an increase in the activity of the sympathetic system and therefore a decrease in the variability of heart rate. 

Resting diastolic blood pressure was measured in two studies and neither of them showed any intra-group or between-group statistical significance (*p* > 0.05). Peak systolic blood pressure was assessed only in one study [47], which did not show any statistically significant difference. Furthermore, resting systolic blood pressure was assessed in three studies [52,55,69], which did not show any statistically significant difference.

In summary, there is a lack of evidence to support that IMT may improve any cardiovascular parameter analyzed in the present review. Further and high-quality studies about cardiovascular parameters are required in the near future.

#### 3.2.8. Biomarkers

The existence of an abnormal immune response seems to play an important role in the HF pathogenesis and progress, including overexpression of pro-inflammatory cytokines such as alpha tumor necrosis factor (TNF- α), interleukin-6 (IL-6), and soluble apoptosis mediators such as soluble Fas (sFas) and soluble Fas ligand (sFasL) [68]. Other biomarkers such as the *N*-terminal pro-brain natriuretic peptide (NT-proBNP) or serum carbohydrate 125 antigen (CA125) may be related to the HF severity, and C-reactive protein (CRP) may be used as an indicator of systemic inflammation [53,69,72].

Six studies [53,54,56,57,68,69] assessed the IMT effects on blood biomarkers, being the NT-proBNP, CA125 and CRP considered as the most studied biomarkers (Table 8). Five studies [53,54,56,57,69] evaluated the effect of IMT on NT-proBNP, although only the study carried out by Adamopoulos et al. [53] showed a significant difference (*p* < 0.05) in favor of IMT, highlighting that this study was the only study that included an aerobic training program. The effect of IMT on CA125 was analyzed in two studies [56,57], showing the absence of significant differences between groups (*p* > 0.05). CRP was evaluated after IMT in three studies [53,54,68], determining a significant difference in favor of IMT according to Adamopoulos et al. [53]. In addition, Laoutaris et al. [68] analyzed IMT modifications on TNF-a, IL-6, sFas and sFasL, and sTNF-RI, although only significant improvements were observed in favor of IMT for sTNF-RI and sFas values. According to Marco et al. [54], improvements were shown for renal function in favor of the sham IMT group, since the glomerular filtration rate was increased (*p* = 0.007), and creatine was decreased (*p* = 0.003).

In conclusion, IMT did not seem to produce key changes in blood biomarkers, although additional studies about sTNF-RI and sFas could be interesting for clinicians and researchers. 

#### 3.2.9. Quality of Life

Quality of life was measured in 15 studies [20,30,31,50,51,52,53,54,55,56,57,60,62,66,67]. Eleven of these studies [20,30,50,52,53,55,56,57,60,62,67] used the Minnesota Living with Heart Failure Questionnaire (MLwHFQ) to measure quality of life, and all of them showed statistically significant improvements (*p* < 0.05) with respect to the control group, except for two studies [60,62]. Three studies [31,51,54] used the SF-36 questionnaire, and none of them showed statistically significant differences (*p* > 0.05). Finally, Johnson et al. [66] used a specific questionnaire for patients with HF that did not show any statistically significant difference. Dall’ago et al. [30] observed that the improvement in MLwHFQ was due to an improvement in physical condition, since psychological section values were similar at baseline of treatment (Table 9).

In summary, IMT improved quality of life under MLwHFQ evaluations but not for SF-36 assessments. Thus, it would be advisable to carry out a study to determine the existence of differences between MLwHFQ and SF-36 in HF patients.

### 3.3. Limitations and Future Studies

Despite data extraction and statistical significances being included in all tables for intra-group and between-group comparisons, some PRISMA checklist recommendations [45], such as methodology quality assessment, risk of bias, heterogeneity analyses and forest plots, were not followed due to insufficient information in part due to the heterogeneity of analyses. The heterogeneity of our study sample hindered our capacity to carry out a systematic review or meta-analysis. Thus, an extensive narrative review was performed including all available scientific evidence about this topic. Indeed, this narrative review analyzed all available experimental studies, including clinical trials, quasi-experimental studies and clinical cases, about IMT in patients with HF. According to all PRISMA recommendations [45], future studies should be carried out following systematic reviews and meta-analysis for specific outcome measurements in order to update strong recommendations to apply IMT interventions in patients who suffered from HF [33,44].

## 4. Conclusions

To date, there is enough evidence to state that IMT produces improvements in respiratory muscle strength and endurance, dyspnea sensation at rest and during exercise, distance covered in gait tests, resistance during exercise, meta-reflex of respiratory muscles, peak VO_2_ and circulatory power. Nevertheless, there is no evidence to determine that IMT could improve VO_2_ anaerobic threshold_,_ cardiovascular parameters and blood biomarkers. In addition, there is a lack of evidence to confirm that IMT may improve quality of life due to controversial findings between MLwHFQ and SF-36 measurements, lung function, VE/VCO_2_, OUES, oxygen pulse, NYHA functional class and limb strength. Finally, mortality or HF hospitalizations were not evaluated, and most studies were not longer than 3 months. According to IMT protocols and study design heterogeneity and mid-term follow-up, further investigations through high-quality long-term randomized clinical trials should be performed to achieve systematic reviews and meta-analysis to support strong evidence for IMT in HF patients.

## Figures and Tables

**Table:** (**a**)

Year and Authors	Subjects Characteristics	Training Protocol
1998. Johnson et al. [66]	Baseline sample *n* = 18 Final sample *n*= 16 M/W: 15/3 FEV1 (%): *N*/A NYHA (II/III): 12/6	Duration: 8 weeks IMT with threshold device F: 7 ×/week. All sessions supervised
	(1) IMT: baseline *n* = 9, final *n* = 8 Age (years): 70 ± 4.6 PI_max_ (cmH_2_O): 70 ± 33	(1) IMT: I: 30% PI_max_, weekly adjusted. T: 15 min, 2 ×/day
	(2) IMT control: baseline *n* = 9, final *n* = 8 Age (years): 63.4 ± 4.5 PI_max_(cmH_2_O): 84 ± 18	(2) IMT control: I: 15% PI_max_ initial, non-readjusted. T: 15 min, 2 ×/day
1999. Weiner et al. [35]	Baseline sample *n* = 20 Final sample *n* = 16 M/W: 18/2 NYHA: II-III	Duration: 12 weeks IMT with threshold device F: 6 ×/week. All sessions supervised T: 30 min for each session
	(1) IMT *n*= 10 Age (years): 66.2 ± 4.6 FEV1 (%): 24.7 ± 1.6 NYHA: 2.3 ± 0.2 PI_max_ (%): 46.5 ± 4.7	(1) IMT: 1st month: I: Initial 15% to get up to 60% PI_max_ gradually Adjusted weekly
	(2) IMT control (baseline *n*= 10, final *n*= 6) Age (years): 63.8 ± 4 FEV1 (%): 22.9 ± 2.4 NYHA: 2.4 ± 0.2 PI_max_ (%): 50.7 ± 4.2	(2) IMT control: Simulated training without resistance
2001. Martínez et al. [63]	Baseline sample *n* = 20 M/W: 16/4	Duration: 6 weeks IMT with threshold device F: 6 ×/week. One weekly session supervised. T: 15 min. 2 ×/day.
	(1) IMT *n* = 11 Age (years): 60 ± 14 FEV1 (%): 28.7 ± 11 NYHA (II/III): 5/6 PI_max_ (cmH_2_O): 78 ± 22	(1) IMT I: 30% PI_max_. PI_max_ weekly adjusted
	(2) IMT control *n* = 9 Age (years): 57 ± 13 FEV1 (%): 27.1 ± 7 NYHA (II/III): 2/7 PI_max_ (cmH_2_O): 72 ± 34	(2) IMT control. I: minimum charge of the device equal to ±10% PI_max_
2004. Laoutaris et al. [67]	Baseline sample *n* = 37 Final sample *n* = 35 NYHA: II (19) y III (18)	Duration: 10 weeks IMT with resistive charge device F: 3 ×/week. All sessions supervised %SMIP readjusted for each session
	(1) IMT: (*n* = 20) H/M: 18/2 Age (years): 57.6 ± 2.3 FEV1 (%): 23.4 ± 1.5 NYHA (II/III): 12/8 PI_max_ (cmH2O): 82.8 ± 5.7	(1) IMT: I: 60% SMIP, readjusted weekly T: 6 efforts for each level: Level 1: 60 s of rest for each 6 inspiratory efforts Level 2: 45 s of rest between series Level 3: 30 s of rest between series Level 4: 15 s of rest between series Level 5: 10 s of rest between series Level 6: 5 s of rest between series. After level 6, a rest of 5 s was maintained up to get respiratory fatigue
	(2) Control IMT: (baseline *n* = 17, final *n* = 15) M/W: 13/2. Age (years): 60 ± 2.6 FEV1 (%): 25.7 ± 2.1 NYHA (II/III): 7/8 PI_max_ (cmH2O): 78.4 ± 6.9	(2) Control by IMT: I: fixed at 15% SMIP T: completed 6 efforts for the 6 levels
2006. Dall’ago et al. [30]	Total sample *n* = 32	Duration: 12 weeks. IMT with threshold device F: 7 ×/week. One weekly session supervised T: 30 min for each session
	(1) IMT *n* = 16 M/W: 10/6 Age (years): 54 ± 3 FEV1 (%): 38 ± 3 NYHA: *N*/A PI_max_ (cmH_2_O): 59.8 ± 2	(1) IMT: IMT: 7 ×/week I: 30% PI_max_, readjusted for each week
	(2) Sham IMT *n* = 16 H/M: 11/5 Age (years): 58 ± 2 FEVI (%): 39 ± 3 NYHA: *N*/A PI_max_ (cmH_2_O): 59.5 ± 2.2	(2) Sham IMT: I: without charge
2007. Laoutaris et al. [68]	Total sample *n* = 38	Duration: 10 weeks. IMT with resistive charge device. F: 3 ×/week. All sessions supervised Readjustment %SMIP foe each session
	(1) High intensity IMT *n* = 15 M/W: 12/3 Age (years): 53 ± 2 FEV1 (%): 28 ± 1 NYHA (II/III) = 10/5 PI_max_ (cmH2O): 79.8 ± 4.7	(1) IMT: I: 60% SMIP, weekly readjusted T: 6 efforts for each level: Level 1: 60 s of rest for each 6 inspiratory efforts Level 2: 45 s of rest between series Level 3: 30 s of rest between series Level 4: 15 s of rest between series Level 5: 10 s of rest between series Level 6: 5 s of rest between series. After level 6, a rest of 5 s was maintained up to get respiratory fatigue
	(2) Low intensity IMT: *n* = 23 M/W: 20/3 Age (years): 59 ± 2 FEV1 (%): 28 ± 1 NYHA (II/III) = 12/11 PI_max_ (cmH2O): 80.2 ± 5	(2) Low intensity IMT: I: fixed at 15% SMIP T: completed 6 efforts in the 6 levels
2008. Laoutaris et al. [69]	Total sample *n* = 23	Duration: 10 weeks IMT with resistive charge device F: 3 ×/week. All sessions supervised Readjustment of %SMIP foe each session.
	(1) High intensity IMT *n* = 14 M/W: 11/3 Age (years): 53.4 ± 2.1 FEV1 (%): 28.9 ± 2.4 NYHA (II/III): 9/5 PI_max_ (cmH2O): 78.5 ± 4.9	(1) IMT: I: 60% SMIP, weekly readjusted T: 6 efforts for each level: Level 1: 60 s of rest for each 6 inspiratory efforts Level 2: 45 s of rest between series Level 3: 30 s of rest between series Level 4: 15 s of rest between series Level 5: 10 s of rest between series Level 6: 5 s of rest between series. After level 6, a rest of 5 s was maintained up to get respiratory fatigue
	(2) Low intensity IMT *n* = 9 M/W: 9/0 Age (years): 57.3 ± 4 FEV1 (%): 28.6 ± 1.9 NYHA (II/III): 6/3 PI_max_ (cmH2O): 84.6 ± 5.9	(2) Low intensity IMT: I: fixed at 15% SMIP. T: completed 6 efforts in the 6 levels
2009. Stein et al. [49]	Total sample *n* = 32 M/W: *N*/A Age (years): *N*/A FEV1 (%):38 ± 3. NYHA: *N*/A PI_max_ (%): <70%	Duration: 12 weeks IMT with threshold device F: 7 ×/week T: 30 min for each session
	(1) IMT *n* = 16	(1) IMT: I: 30% PI_max_, weekly readjusted
	(2) Sham IMT *n* = 16	(2) Sham IMT: I: Without charge.
2009. Padula et al. [31]	Total sample *n* = 32 FEV1 (%): <45	Duration: 12 weeks
	(1) IMT *n* = 15 M/W: 5/10 Age (years): 76 (51–89) PI_max_ (cmH2O): 48 ± 25 NYHA (II/III): 5/7	(1) IMT: IMT with threshold device F: 6–7 ×/week I: 30% PI_max_, readjusted each 3 weeks T: 10–20 min/day
	(2) Control group *n* = 17 M/W: 7/10 Age (years): 73 (32–95) PI_max_ (cmH2O): 52 ± 27 NYHA (II/III): 9/6	(2) Control: Education about auto-efficacy, anatomy and physiology
2011. Bosnak-Guclu et al. [51]	Total sample *n* = 30	Duration: 6 weeks IMT with threshold device F: 7 ×/week. One session weekly supervised T: 30 min for each session
	(1) IMT *n* = 16 M/W: 12/4 Age (years): 70 ± 8 FEV1 (%): 33 ± 7 NYHA (II/III): 11/5 PI_max_ (cmH_2_O): 62 ± 33	(1) IMT group: I: 40% PI_max_, readjusted each week
	(2) Sham IMT *n* = 14 M/W: 12/2 Age (years): 66 ± 11 FEVI (%): 36 ± 8 NYHA (II/III): 9/5 PI_max_ (cmH_2_O): 78 ± 35	(2) Sham IMT group: I: fixed at 15% PI_max_
2012. Mello et al. [55]	Total sample *n*= 27 NYHA: II	Duration: 12 weeks
	(1) IMT *n* = 15 M/W: 9/6 Age (years): 54.3 ± 2 FEV1 (%): 33.6 ± 2.3 PI_max_ (cmH_2_O): 56.1 ± 2.3	(1) IMT: IMT with threshold device I: 30% PImax, weekly readjusted F: 7 ×/week. One session weekly supervised T: 10 min × 3/day.
	(2) Control group *n* = 12 M/W: 5/7 Age (years): 53.3 ± 2 FEV1 (%): 37.6 ± 1.6 PI_max_ (cmH_2_O): 56.2 ± 2.1	(2) Control group Usual care
2013. Marco et al. [54]	Total sample *n* = 22	Duration: 4 weeks IMT with threshold device F: 7 ×/week. One session weekly supervised IMT was performed 5 × 10 with 1/2 min of rest between series at 2 times per day
	(1) High intensity IMT *n* = 11 M/W: 7/4 Age (years): 68.5 ± 8.9 FEV1 (%): 38.3 ± 16 NYHA (II/III): 8/3 PI_max_ (cmH_2_O): 55.1 ± 23.6	(1) High intensity IMT: I: 100% of 10 RM, weekly adjusted
	(2) Sham IMT *n* =11 M/W: 10/1 Age (years): 70.1 ± 10.1 FEV1 (%): 35.5 ± 17.5 NYHA (II/III): 9/2 PI_max_ (cmH_2_O): 58.1 ± 24.3	(2) Sham IMT: I: 10 cmH_2_O was weekly increased up to 2.5 cmH_2_O
2014. Palau et al. [56]	Total sample *n* = 26	Duration: 12 weeks IMT with threshold device
	(1) IMT *n* = 14 M/W: 7/7 Age (years): 68 (60–76) FEV1 (%): 69 (63–77) NYHA (II/III-IV): 5/9 PI_max_ (cmH_2_O): 70 (55.7–84)	(1) IMT: I: 25/30% of PI_max_ weekly readjusted F: 7 ×/week. One session weekly supervised T: 20 min ×2/day
	(2) Control group *n* = 12 M/W: 6/6 Age (years): 74 (73–77) FEV1 (%): 76 (68–83) NYHA (II/III-IV): 3/9 PI_max_ (cmH_2_O): 68 (60.5–88.5)	(2) Control group: Usual care
2017. Moreno et al. [20]	Total sample *n* = 26	Duration: 8 weeks IMT with threshold or resistive charge device
	(1) IMT *n* = 13 M/W: 8/5 Age (years): 61 ± 14 FEV1 (%): 35 ± 9 NYHA (II/III): 6/7 PI_max_ (cmH_2_O): 60 ± 13	(1) IMT: I: 30% PI_max_, weekly readjusted. F: 6 ×/week. One session weekly supervised T: 30 min for each session
	(2) Control group *n* = 13 M/W: 8/5 Age (years): 60 ± 13 FEV1 (%): 37 ± 6 NYHA (II/III): 7/6 PI_max_ (cmH_2_O): 60 ± 16	(2) Control group: Without intervention

F = Frequency; IMT = Inspiratory muscle training; M = men, *N*/A = No data available; PI_max_ = Maximum inspiratory pressure; SMIP = Sustained maximum inspiratory pressure; T = Time; x = repetitions; W = Women.

**Table:** (**b**)

Year and Authors	Subjects Characteristics	Training Protocol
2009. Winkelmann et al. [50]	Total sample *n* = 38 NYHA: *N*/A	Duration: 12 weeks All groups carried out AT which consisted of: Static bicycle training with a cadence of 60 rpm I: RPE of 5/10 T: initial 20 min, and 5 min added for each 2 weeks up to get 45 min
	(1) AT + IMT baseline *n* = 19, final *n* = 12 M/W: 4/8 Age (years): 54 ± 12 FEV1 (%): 39 ± 12 PI_max_ (cmH2O): 57 ± 12	(1) IMT + AT: IMT with threshold device F: 7 ×/week. One session weekly supervised I: 30% PI_max_, readjusted for each week T: 30 min per session
	(2) AT baseline *n* i = 19, final *n* = 12 M/W: 7/5 Age (years): 59 ± 9 FEV1 (%): 34 ± 11 PI_max_ (cmH2O): 56 ± 13	(2) AT: Isolated AT was performed
2013. Laoutaris et al. [52]	Total sample *n* = 27	Duration: 12 weeks F: 3 ×/week. All sessions supervised AT was carried out by both groups at 70/80% of maximum HR during static bicycle
	(1) ARIS *n* = 13 M/W: 10/3 Age (years): 57.1 ± 11 FEV1 (%): 27.8 ± 8 NYHA (II/III): 6/7 PI_max_ (cmH2O): 75.3 ± 11	(1) ARIS: AT began with 20 min and was minimum increased 1 min for each session up to get 30 min. RT consisted of 3 × 12 quadriceps bench strengthening exercises at 50% of 1 RM (adjusted each 2 weeks, 4 × 12 exercises performing elbow flexion, abduction and elbow flexion with weights from 1 to 2 kg IMT was performed with a resistive charge device; rest duration was decreased between inspiratory efforts according to the patients’ clinical course. I: 60% SMIP, adjusted in each session T: 1 h 15 min for the total session
	(2) AT *n* = 14 M/W: 12/2 Age (years): 58.6 ± 8 FEVI (%): 30.6 ± 5.4 NYHA (II/III): 8/6 PI_max_ (cmH2O): 79 ± 9.1	(2) AT: AT was expanded up to 45 min. T: 55 min for the total session duration
2014. Adamopoulos et al. [53]	Total sample *n*= 43	Duration: 12 weeks F: 3 ×/week. All sessions supervised. AT was carried out by both groups at 70%/80% of the maximum HR in static bicycle during 45 min.
		IMT was performed with a resistive charge device. Both groups carried out the following protocol. Six efforts were performed for each level Level 1: 60 s of rest for each 6 inspiratory efforts Level 2: 45 s of rest between series Level 3: 30 s of rest between series Level 4: 15 s of rest between series Level 5: 10 s of rest between series Level 6: 5 s of rest between series. After level 6, a rest of 5 s was maintained up to get 30 min of IMT.
	(1) AT + IMT *n* = 21 M/W: 19/2 Age (years): 57.8 ± 11.7 FEV1 (%): 27.7 ± 6.7 NYHA (II/III): 9/12 PI_max_ (cmH2O): 81.9 ± 21.5	(1) AT/IMT: I: 60% SMIP, adjusted for each session
	(2) AT+ Sham IMT *n* = 22 M/W: 17/5 Age (years): 58.3 ± 13.2 FEV1 (%): 30.1 ± 5 NYHA (II/III): 12/10 PI_max_ (cmH2O): 79.1 ± 19.4	(2) AT/IMT simulated: I: 10% SMIP, adjusted for each session
2017. Kawauchi et al. [60]	Total sample *n* = 35	Duration: 8 weeks. IMT was performed with a threshold device F: 7 ×/week. One session supervised each 15 days, and IMT progressions and RT were performed each 15 days. IMT lasted 30 min for each session in both groups. RT was performed 1 x10 for each exercise (elbow flexion and extension, shoulder flexion and abduction, hip flexion, extension and abduction, plantar and dorsal flexion) during the first 2 weeks and 2 series of 10 repetitions during the rest 6 weeks
	(1) MIPRT *n* = 13 M/W: 8/5 Age (years): 56 ± 7 FEV1 (%): 28 ± 5 NYHA (II/III): 5/8 PI_max_ (cmH2O): 70 ± 14	(1) MIPRT: IMT was performed at an intensity of 30% PI_max_ RT at an intensity of 50% 1 RM
	(2) LIPRT *n* = 13 M/W: 6/7 Age (years): 54 ± 10 FEV1 (%): 30 ± 6 NYHA (II/III): 6/7 PI_max_ (cmH2O): 72 ± 20	(2) LIPRT: IMT was performed at an intensity of 15% PI_max_ RT with weights of 0.5 kg
	(3) Control group *n* = 9 M/W: 5/4 Age (years): 56 ± 7 FEV1 (%): 29 ± 7 NYHA (II/III): 5/4 PI_max_ (cmH2O): 74 ± 24	(3) Control group: Without intervention
2018. Palau et al. [57]	Total sample *n* = 59	Duration: 12 weeks IMT with threshold device
	(1) IMT *n* = 15 M/W: 7/8 Age (years): 75 ± 10 FEV1 (%): 70 ± 9 NYHA (II/III): 12/3 PI_max_ (cmH2O): 58 ± 20	(1) IMT: F: 7 ×/weeks. One session supervised I: 25/30% PI_max_, weekly readjusted T: 20 min x2/day.
	(2) FES *n* = 15 M/W: 6/8 Age (years): 72 ± 9 FEV1 (%): 68 ± 11 NYHA (II/III): 10/5 PI_max_ (cmH2O): 53 ± 16	(2) FES: F: 2 ×/week. All sessions supervised T: 45 min for each session FES consisted of functional electric stimulation in lower limbs with a low frequency biphasic electric current
	(3) IMT + FES *n* = 16 M/W: 8/8 Age (years): 73 ± 10 FEV1 (%): 63 ± 11 NYHA (II/III): 11/5 PI_max_ (cmH2O): 59 ± 26	(3) IMT + FES: IMT + FES was applied during 12 weeks
	(4) Control group *n* = 13 M/W: 4/9 Age (years): 75 ± 9 FEVI1(%): 66 ± 8 NYHA (II/III): 8/5 PI_max_ (cmH2O): 58 ± 25	(4) Control group: Usual care
2019. Hornikx et al. [62]	Total sample *n* = 20 NYHA: *n*/A	Duration: 3 months IMT was carried out with a resistive charge device F: 3 ×/week. All sessions supervised
	(1) RHIIT *n* = 10 M/W: 5/5 Age (years): 64 ± 8 FEV1 (%): 30 ± 14 PI_max_ (cmH2O): 64 ± 27	(1) RHIIT: IMT, RT and HIIT were performed IMT: F: 7 ×/week. I: 50% PI_max_, weekly readjusted This protocol comprised 30 repetitions at 2 time per day RT: 2 × 10 at 65% of 1 RM leg press exercise, increasing weights according to subjective patients’ sensations HIIT: 5 series of 3 min at 80% maximum charge work (Wpeak) and between series an active recuperation of 3 min was added (40% Wpeak)
	(2) SP *n* = 10 M/W: 6/4 Age (years): 58 ± 11 FEV1 (%): 31 ± 14 PI_max_ (cmH_2_O): 89 ± 28	(2) SP: SP comprised active 60 min. Training intensity began at 50% Wpeak and was progressively increased during 3 months up to 98% Wpeak in the last week. Each session included a 3 min warm-up, followed by 2 × 7 min cycling, 2 × 7 min walking in the treadmill, followed by rowing exercise, steps and arm ergometry during 12 min. Finally, calisthenics of all large muscle groups were added during 20 min
2019. Hossein Pour et al. [61]	Total sample *n* = 84	Duration: 6 weeks IMT was performed with threshold device F: 7 ×/week. One session weekly supervised T: 30 min for each session
	(1) IMT *n* = 42 M/W: 23/19 Age (years): 56 ± 9.4 FEV1 (%): 33.7 ± 6.1 NYHA (II/III/IV): 15/23/4 PI_max_ (cmH2O): 59 ± 42.5	(1) IMT: I: 40% PI_max_, weekly readjusted
	(2) Sham IMT *n* = 42 M/W: 21/21 Age (years): 57.3 ± 9 FEV1 (%): 32.5 ± 4.4 NYHA (II/III/IV): 17/19/6 PI_max_ (cmH2O): 61.2 ± 72.3	(2) Sham IMT: I: fixed at 10% PI_max_

ARIS = combined AT/RT/IMT; AT = Aerobic training; RT = Resistance training; F = Frequency; FES = Functional electrical stimulation; HR = Heart rate; HIIT = high intensity interval training; I = Intensity; IMT = Inspiratory muscle training; LIPRT = Low intensity (IMT) plus resistance training; M = Men; MIPRT = Moderate intensity (IMT) plus resistance training; *N*/A = No data available; RPE = PI_max_ = Maximum inspiratory pressure; Rate of perceived exertion; RT = Resistance training; SMIP = Sustained maximum inspiratory pressure; RHIIT = Resistance training supplemented HIIT; RM = One-repetition maximum; SP = Standard protocol; T = Time; x = repetitions; W = Women.

**Table:** (**c**)

Year and Authors	Subjects Characteristics	Training Protocol
1995. Mancini et al. [64]	Baseline sample *n* = 14 Final sample *n* = 8 NYHA (I/II/III/IV): 2/2/6/4 PI_max_ (cmH2O): 64 ± 31	Duration: 3 months F: 3 ×/week. All sessions were supervised T: 90 min per session
		Protocol: (1) Isocapnic hyperpnea with 20 min per session
	(1) Training group *n*= 8 Age (years): 56 ± 15 FEV1 (%): 20 ± 8 NYHA: 2.8 ± 1	(2) IMT with threshold device: T: 20 min I: 30% PI_max_ F: 3 ×/week supervised, 2 x/day non-supervised, 15 min. Each 2 weeks training intensity was increase at +5 cmH2O.
	(2) Control group (loss to follow-up) *n* = 6 Age (years): 55 ± 15 FEV1 (%): 24 ± 10 NYHA: 2.3 ± 1.2	(3) 10 repetitions of maximum inspiration and 10 repetitions of maximum expiration. These were maintained for 10 s and rest for 15 s between repetitions
		(4) Rehabilitation respiratory exercises, 8 repetitions for exercise/session.
1997. Cahalin et al. [65]	Baseline sample *n* =14, M/W: 12/2 Final sample *n* = 8 Age (years): 52 ± 8.5 FEV1 (%): 23 ± 13 NYHA: 3.6 ± 0.6 PI_max_ (%): 44 ± 15	Duration: 8 weeks
		(1) IMT with threshold device: T: 5 to 15 min. Initially 5 min and progressively increasing to 15 min for each session I: 20% PI_max_. PI_max_ weekly readjusted F: 3 times daily. 2 times weekly. Supervised sessions
2008. Chiappa et al. [48]	Total sample *n* = 28	Duration: 4 weeks IMT with threshold device
	(1) IMT *n* = 18 M/W: 12/6 Age (years): 57 ± 11 FEV1 (%): 24 ± 3 NYHA (I-II/III-IV): 10/8 PI_max_ (cmH2O): 60 ± 8	(1) IMT F: 7 ×/week. One session weekly supervised I: 30% PI_max_, weekly readjusted T: 30 min per session
	(2) Control group *n* = 10 Healthy subjects M/W: 8/2 Age: 38 ± 12. PI_max_ (cmH2O): 153 ± 26	(2) Control group Without intervention
2019. Palau et al. [58]	Total sample *n* = 45 M/W: 24/21 Age (years): 73 (68–77) FEV1 (%): 67.8 ± 10.3 NYHA (II/III-IV): 29/16 PI_max_ (cmH2O): 61.3 (51.3–72.5)	Duration: 12 weeks IMT with threshold device I: 25/30% PI_max_, readjusted at 7/10 days. F: 7 ×/week. One session supervised at 7/10 days T: 20 min ×2/day.
2019. Taya et al. [59]	Total sample *n* = 1 M/W: 1/0 Age (years): 55 FEV1 (%): 21 NYHA: *N*/A PI_max_ (cmH2O): 45	Duration: 7 weeks AT was carried out with an ergometer at 15–20 W during 7–15 min and 1–2 series were performed; training and charge duration were progressively increased. IMT: Training performed with a resistive charge device F: 7 ×/week. One session weekly supervised I: 20% PI_max_ weekly readjusted Training protocol comprised 2 series of 30 repetitions

AT = Aerobic training; F = Frequency; I = Intensity; IMT = Inspiratory muscle training; *N*/A = No data available; M = men; PI_max_ = Maximum inspiratory pressure; T = Time; x = repetitions; W = Women.

**Table:** (**a**)

Studies	Groups	PI_max_ (cmH_2_O, kPa o %) *				PE_max_ (cmH_2_O o %) *				Inspiratory Muscle Resistance				
		Pre	Post	*p*-Value *	*p*-Value **	Pre	Post	*p*-Value *	*p*-Value **	A.I.	Pre	Post	*p*-Value *	*p*-Value **
Mancini, 1995	Training *n* = 8	64 ± 31	88 ± 34	<0.01	<0.05	94 ± 30	152 ± 40	<0.001	<0.05		-	-	-	-
	Ctl. *n* = 6	*N*/A	*N*/A	*N’S*		*N*/A	*N*/A	*N’S*			-	-	-	
Cahalin, 1997	IMT *n* = 8	51 ± 21	63 ± 23	0.0001	-	85 ± 22	96 ± 19	0.0001	-		-	-	-	-
Johnson, 1998	IMT *n* = 8	70 ± 33	+25.4 ± 11.2	*n*/A	0.04	-	-	-	-		-	-	-	-
	C.IMT *n* = 8	84 ± 18	+12.3 ± 12.1	*N*/A		-	-	-			-	-	-	
Weiner, 1999	IMT *n* = 10	46.5 ± 4.5	63.6 ± 4	<0.005	*N*/A	82.1 ± 6.1	*N*/A	*N’S*	*N*/A	PM_peak_/PI_max_ were calculated and expressed as %	47.8 ± 3.6	67.7 ± 1.7	<0.005	*N*/A
	C.IMT *n* = 6	50.7 ± 4.2	*N*/A	*N’S*		80.8 ± 5.7	*N*/A	*N’S*			45.6 ± 3.5	*N*/A	*N’S*	
Martínez, 2001	IMT *n* = 11	78 ± 22	99 ± 22	<0.01	*N*/A	-	-	-	*N*/A	Sustained PI_max_ (SMIP) was calculated for 2 min (cmH_2_O)	63 ± 18	90 ± 22	<0.01	*N*/A
	C.IMT *n* = 9	72 ± 34	83 ± 30	<0.05		-	-	-			58 ± 30	69 ± 30	<0.05	
Laoutaris, 2004	IMT *n* = 20	82.8 ± 5.7	111.9 ± 6.8	0.000	*N*/A	-	-	-	-	SMIP was measured for a variable duration (cmH2O/s^10^−1^)	367360 ± 41111	527822 ± 51358	0.000	*N*/A
	C.IMT *n* = 15	78.4 ± 6.8	86.6 ± 6.3	0.03		-	-	-			271995 ± 30308	209065 ± 34896	0.003	
Dall’ago, 2006	IMT *n* = 16	59.5 ± 2.2%	*N*/A	*N*/A	<0.01	-	-	-	-	SMIP measured for 1 min by an incremental test (Pth_max_) (kPa)	3.2 ± 0.5	3.8 ± 0.5	<0.05	<0.001
	S.IMT *n* = 16	59.8 ± 2%	*N*/A	*N*/A		-	-	-			3.1 ± 0.5	3.2 ± 0.6	*N’S*	
Laoutaris, 2007	H.IMT *n* = 15	79.8 ± 4.7	105.1 ± 4.9	<0.001	*N’S*	-	-	-	-	SMIP measured for a variable duration (cmH2O/s^10^3^)	312 ± 27	504 ± 40	<0.001	<0.01
	L.IMT *n* = 23	80.2 ± 5	90.3 ± 5.9	<0.01		-	-	-			286 ± 27	257 ± 35	*N’S*	
Chiappa, 2008	IMT *n* = 18	60 ± 8	103 ± 16	<0.05	-	-	-	-	-		-	-	-	-
Laoutaris, 2008	H.IMT *n* = 14	79 ± 5	105 ± 5.3	<0.05	*N’S*	-	-	-	-	SMIP measured for a variable duration (cmH2O/s^10^3^)	308 ± 28	511 ± 42	<0.05	<0.05
	L.IMT *n* = 9	82.2 ± 8.7	97.6 ± 11.3	<0.05		-	-	-			*N*/A	*N*/A	*N’S*	
Padula, 2009	IMT *n* = 15	48.7 ± 25.7	78.5 ± 37.1	*N*/A	<0.0001	-	-	-	-		-	-	-	-
	Ctl. *n* = 17	52.3 ± 27.3	52.6 ± 28.3	*N*/A		-	-	-			-	-	-	
Stein, 2009	IMT *n* = 16	5.9 ± 0.9 kPa	12.7 ± 0.9 kPa	<0.001	*N*/A	-	-	-	-		-	-	-	-
	S.IMT *n* = 16	*N*/A	*N*/A	*N’S*		-	-	-			-	-	-	
Winkelmann, 2009	ATIMT, *n* = 12	57 ± 12	*N*/A	<0.05	<0.01	79 ± 31	123 ± 31	<0.001	<0.05	Pth_max_ (cmH_2_O)	28 ± 6	41 ± 2	*N*/A	<0.001
	AT *n* = 12	56 ± 13	*N*/A	<0.05		74 ± 23	108 ± 27	*N*/A			29 ± 6	36 ± 3	*N*/A	
Bosnak-Guclu, 2011	IMT *n* = 16	62 ± 33.6	97.1 ± 32.6	<0.001	<0.001	102.6 ± 55.2	125.1 ± 56.2	<0.001	0.009		-	-	-	-
	S.IMT *n* = 14	78.6 ± 36	90.9 ± 30.2	0.001		115.9 ± 43.2	124.7 ± 50.4	0.026			-	-	-	
Mello, 2012	IMT *n* = 15	59.2 ± 4.9	87.5 ± 6.5	0.001	<0.05	-	-	-	-		-	-	-	-
	Ctl. *n* = 12	63.2 ± 5.3	67.8 ± 5.8	*N’S*		-	-	-			-	-	-	
Laoutaris, 2013	ARIS *n* = 13	75.3 ± 11	102 ± 19	<0.001	*N’S*	-	-	-	-	SMIP measured for a variable duration (cmH2O/s^10^3^)	310 ± 27	413 ± 24	<0.001	<0.001
	AT *n* = 14	79 ± 9.1	83.5 ± 9.7	0.02		-	-	-			306 ± 21	307 ± 23	N	
Marco 2013	H.IMT *n* = 11	56.1 ± 19.9	88.2 ± 21.3	*N*/A	0.001	-	-	-	-	10 RM of maximum inspiratory charge measured (cmH_2_O)	34.4 ± 12.8	59.4 ± 17.5	-	<0.001
	L.IMT *n* = 11	56.1 ± 15.6	70.8 ± 16.4	*N*/A		-	-	-			33 ± 12.1	39 ± 10.1	-	
Adamopoulos, 2014	AT + IMT *n* = 21	81.9 ± 21.5	100.7 ± 23	<0.001	*N’S*	-	-	-	-	SMIP measured for a variable duration (cmH2O/s^10^3^)	343 ± 120	521 ± 146	<0.001	0.02
	ATSIMT *n* = 22	79.2 ± 19.4	85.1 ± 25	0.02		-	-	-			330 ± 125	350 ± 159	*N’S*	
Palau, 2014	IMT *n* = 14	70 (55.7–84)	133 (92–190)	<0.001	*N*/A	-	-	-	-		-	-	-	-
	Ctl. *n* = 12	68 (61–89)	68 (58–90)	*N’S*		-	-	-			-	-	-	
Kawauchi, 2017	MIPRT *n* = 13	70 ± 14	92 ± 26	<0.05	<0.05 ***	90 ± 32	114 ± 32	<0.05	<0.05 ***		-	-	-	-
	LIPRT *n* = 13	72 ± 20	89 ± 28	<0.05	<0.05 ***	100 ± 30	107 ± 33	<0.05	*N’S*		-	-	-	-
	Ctl. *n* = 9	74 ± 24	69 ± 25	*N’S*	-	98 ± 31	93 ± 27	*N’S*	-		-	-	-	-
Moreno, 2017	IMT *n* = 15	60 ± 13	*N*/A	<0.001	<0.001	-	-	-	-		-	-	-	-
	Ctl. *n* = 13	60 ± 16	*N*/A	*N’S*		-	-	-			-	-	-	
Hornikx, 2019	RHIIT *n* = 10	64 ± 27	+44.9 ± 29.9	<0.01	<0.01	-	-	-	-		-	-	-	-
	SP *n* = 10	89 ± 28	−0.56 ± 19.4	*N’S*		-	-	-			-	-	-	
Palau, 2019	IMT *n* = 45	61 (51–73)	97 (82–150)	<0.001	-	-	-	-	-		-	-	-	-
Taya, 2019	IMT + AT *n* = 1	54.9%	102.3%	*N*/A	-	48.8%	62.7%	*N*/A	-		-	-	-	-

* % refers to % predicted according to age and sex, kPa refers to this measurement units, and if no unit was marked, cmH_2_O was considered. PM_peak_: sustained pressure during at least 60 s with the heaviest charge. *p*-value *: showed intra-group statistical significance. *p*-value **: showed between-groups statistical significance. *p*-value ***: *p*-value of intervention group vs. control at the same time point. *N’S*: non-significant (*p* > 0.05) *N*/A: No data available, or available tables did not permit to determine these values correctly; A.I. = Additional information; ARIS = combined AT/RT/IMT; AT = Aerobic training; ATIMT = AT + IMT; ATSIMT: AT + S.IMT; C.IMT = control group IMT; Ctl.= control group; H.IMT = high intensity IMT; L.IMT = low intensity IMT; IMT = Inspiratory muscle training; PE_max_ = Maximum expiratory pressure; PI_max_ = Maximum inspiratory pressure; Pth_max_ = Pressure threshold maximum; RT = Resistance training; S.IMT = sham IMT; SMIP = Sustained maximum inspiratory pressure; RM = One-repetition maximum.

**Table:** (**b**)

Studies	Groups	FVC (% Predicted or L)				FEV1(% Pred o L)				FEV1/FVC			
		Pre	Post	*p*-Value *	*p*-Value **	Pre	Post	*p*-Value *	*p*-Value **	Pre	Post	*p*-Value *	*p*-Value **
Mancini, 1995	Training *n* = 8	-	-	-	-	*N*/A	2.3 ± 0.7 L	*N’S*	*N’S*	*N*/A	72 ± 8	*N’S*	*N’S*
	Ctl. *n* = 6	-	-	-		*N*/A	3.1 ± 1.4 L	*N’S*		*N*/A	73 ± 10	*N’S*	
Weiner, 1999	IMT *n* = 10	3.14 ± 0.2 L	3.37 ± 0.2 L	<0.05	*N*/A	2.46 ± 0.2 L	*N*/A	*N’S*	*N*/A	-	-	-	-
	C.IMT *n* = 6	3.02 ± 0.6 L	*N*/A	*N*/A		2.33 ± 0.2 L	*N*/A	*N’S*		-	-	-	
Laoutaris, 2004	IMT *n* = 20	92.4 ± 4.2%	98.1 ± 4.2%	*N’S*	*N*/A	90.5 ± 4.5%	91.6 ± 5%	*N’S*	*N*/A	78 ± 2	74 ± 2	0.006	*N*/A
	C.IMT *n* = 15	87.9 ± 3.1%	89.9 ± 3.3%	*N’S*		83.2 ± 4.9%	81.3 ± 4.4%	*N’S*		77.1 ± 3.2	73.2 ± 3	*N’S*	
Dall’ago, 2006	IMT *n* = 16	85.3 ± 13.4%	84.8 ± 15.2%	*N’S*	*N’S*	83.7 ± 14.5%	82.4 ± 15.1%	*N’S*	*N’S*	-	-	-	-
	S.IMT *n* = 16	84.7 ± 8.8%	83 ± 9.5%	*N’S*		90.1 ± 12.6%	90.1 ± 12.6%	*N’S*		-	-	-	
Laoutaris, 2007	H.IMT *n* = 15	96 ± 3.3%	98.9 ± 3.9%	<0.05	<0.05	91.3 ± 4.1%	93.3 ± 4.1%	*N’S*	<0.05	76.7 ± 1.8	76.4 ± 1.9	*N’S*	*N’S*
	L. IMT *n* = 23	85.8 ± 2.6%	88 ± 2.6%	*N’S*		80.1 ± 3.8%	79.9 ± 3.7%	*N’S*		75.8 ± 2.5	72.7 ± 2.5	0.05	
Bosnak-Guclu, 2011	IMT *n* = 16	92.1 ± 15%	102.5 ± 15.9%	0.001	*N’S*	84.6 ± 16%	89.6 ± 14.6%	0.024	*N’S*	71.2 ± 10.3	69 ± 11.3	*N’S*	0.02
	S.IMT *n* = 14	91.6 ± 14.7%	97.6 ± 15.3%	0.023		86.8 ± 20.8%	89.7 ± 20%	*N’S*		71.8 ± 8.3	74.3 ± 5.9	*N’S*	
Adamopoulos, 2014	AT + IMT *n* = 21	85.4 ± 16.1%	84.7 ± 20%	*N’S*	*N’S*	85.1 ± 14.9%	82.5 ± 21.4%	*N’S*	*N’S*	91 ± 12.5	85.9 ± 19.7	*N’S*	*N’S*
	AT + S.IMT *n* = 22	89.9 ± 20.4%	94.5 ± 18.6%	*N’S*		84.8 ± 18.3%	90.4 ± 18%	*N’S*		89.4 ± 15.2	91.6 ± 9.7	*N’S*	
Kawauchi, 2017	MIPRT *n* = 13	76 ± 13%	76 ± 10%	*N’S*	*N’S* ***	71 ± 16%	73 ± 14%	*N’S*	*N’S* ***	75 ± 7	76 ± 8	*N’S*	*N’S* ***
	LIPRT *n* = 13	78 ± 19%	79 ± 18%	*N’S*	*N’S* ***	74 ± 22%	73 ± 19%	*N’S*	*N’S* ***	76 ± 7	75 ± 7	*N’S*	*N’S* ***
	Ctl. *n* = 9	77 ± 9%	75 ± 11%	*N’S*	-	68 ± 13%	66 ± 12%	*N’S*	-	70 ± 9	70 ± 8	*N’S*	-

*p*-value *: showed intra-group statistical significance. *p*-value **: showed between-groups statistical significance. *p*-value ***: *p*-value of intervention group vs. control at the same time point. *N*/A: No data available, or available tables did not permit to determine these values correctly. *N’S*: non-significant (*p* > 0.05). AT = Aerobic training; ATSIMT = AT +S.IMT; Ctl. = control group; C.IMT = control IMT; H.IMT = high intensity IMT; IMT = Inspiratory muscle training; LIPRT = Low intensity (IMT) plus resistance training; L.IMT = low intensity IMT; MIPRT = Moderate intensity (IMT) plus resistance training; S.IMT = Sham IMT.

**Table:** (**a**)

Study	Groups	Borg Scale				MMRC				Mahler Index				Dyspnea Index			
		Pre	Post	*p*-Value *	*p*-Value **	Pre	Post	*p*-Value *	*p*-Value **	Pre	Post	*p*-Value *	*p*-Value **	Pre	Post	*p*-Value *	*p*-Value **
Mancini, 1995	Training *n* = 8	11 ± 4 (6)	10 ± 2 (6)	*N’S*	*N*/A	-	-	-	-	-	-	-	-	-	-	-	-
	Ctl. *n* = 6	10.3 ± 2.9 (6)	10.7 ± 2.3 (6)	*N’S*		-	-	-		-	-	-		-	-	-	
Cahalin, 1997	IMT *n* = 8	2 ± 0.7 (R)	1.3 ± 0.05 (R)	0.0001	-	-	-	-	-	-	-	-	-	-	-	-	-
		3.6 ± 0.5 (E)	2.6 ± 0.6 (E)	0.003		-	-	-		-	-	-		-	-	-	
Johnson, 1998 *	IMT *n* = 8	8.9 ± 1.9 (S)	−1.2 (S)	*N*/A	*N’S* (S) *	-	-	-	-	-	-	-	-	-	-	-	-
		10.7 ± 1.9 (*n*)	−0.5 (*n*)	*N*/A	*N’S* (*N*) *	-	-	-		-	-	-		-	-	-	
		12.8 ± 2.3 (F)	−1.3 (F)	*N*/A	*N’S* (F) *	-	-	-		-	-	-		-	-	-	
	C.IMT *n* = 8	8.8 ± 1.9 (S)	+0.4 (S)	*N*/A		-	-	-		-	-	-		-	-	-	
		10.4 ± 1.8 (*n*)	−0.1 (*n*)	*N*/A		-	-	-		-	-	-		-	-	-	
		13.1 ± 2.4 (F)	+0.2 (F)	*N*/A		-	-	-		-	-	-		-	-	-	
Weiner, 1999	IMT *n* = 10	-	-	-	-	-	-	-	-	-	-	-	-	1.70 ± 0.2	2.70 ± 0.2	<0.005	*N*/A
	C.IMT *n* = 6	-	-	-		-	-	-		-	-	-		1.75 ± 0.2	*N*/A	*N’S*	
Martínez, 2001	IMT *n* = 11	-	-	-	-	-	-	-	-	6.2 ± 2	2.7 ± 1.8	*N*/A	*N*/A	-	-	-	-
	C.IMT *n* = 9	-	-	-		-	-	-		5 ± 2	2.8 ± 1.8	*N*/A		-	-	-	
Laoutaris, 2004 *	IMT *n* = 20	14.2 ± 0.5 (T)	12.8 ± 0.6 (T)	0.000	*N*/A	-	-	-	-	-	-	-	-	-	-	-	-
		10.5 ± 0.7 (6)	9 ± 0.5 (6)	0.001		-	-	-		-	-	-		-	-	-	
	C.IMT *n* = 15	14.3 ± 0.5 (T)	14.4 ± 0.5 (T)	*N’S*		-	-	-		-	-	-		-	-	-	
		12.7 ± 0.8 (6)	12.6 ± 0.8 (6)	*N’S*		-	-	-		-	-	-		-	-	-	
Dall’ago, 2006	IMT *n* = 16	3.7 ± 2.0 (6)	1.5 ± 1.4 (6)	*N/A*	<0.002	-	-	-	-	-	-	-	-	-	-	-	-
	S.IMT *n* = 16	3.1 ± 1.3 (6)	3.0 ± 1.4 (6)	*N/A*		-	-	-		-	-	-		-	-	-	
Laoutaris, 2007 *	H.IMT *n* = 15	9.2 ± 0.4 (6)	8 ± 0.4 (6)	*<0.01*	<0.001	-	-	-	-	-	-	-	-	-	-	-	-
	L.IMT *n* = 23	11.8 ± 0.6 (6)	11.5 ± 0.6 (6)	*N’S*		-	-	-		-	-	-		-	-	-	
Laoutaris, 2008 *	H.IMT *n* = 14	18.1 ± 0.1 (C)	17.6 ± 0.2 (C)	0.02	0.05	-	-	-	-	-	-	-	-	-	-	-	-
	L.IMT *n* = 9	17.6 ± 0.2 (C)	17.9 ± 0.3 (C)	*N’S*		-	-	-		-	-	-		-	-	-	
Padula, 2009	IMT *n* = 15	*N*/A	*N*/A	*N*/A	*N*/A	-	-	-	-	-	-	-	-	-	-	-	-
	Ctl. *n* = 17	*N*/A	*N*/A	*N*/A		-	-	-		-	-	-		-	-	-	
Bosnak-Guclu, 2011	IMT *n* = 16	-	-	-	-	2.27 ± 0.88	1.07 ± 0.79	<0.001	<0.001	-	-	-	-	-	-	-	-
	S.IMT *n* = 14	-	-	-		1.93 ± 0.92	1.71 ± 0.83	0.024		-	-	-		-	-	-	
Laoutaris, 2013 *	ARIS *n* = 13	17.8 ± 0.6 (C)	17.3 ± 0.9 (C)	*N’S*	0.03	-	-	-	-	-	-	-	-	-	-	-	-
	AT *n* = 14	18.1 ± 0.5(C)	17.8 ± 0.7(C)	*N’S*		-	-	-		-	-	-		-	-	-	
Marco, 2013	H.IMT *n* = 11	-	-	-	-	2.1 ± 1	−0.8 ± 1.39	*N*/A	*N’S*	-	-	-	-	-	-	-	-
	S.IMT *n* = 11	-	-	-		1.6 ± 1.03	−0.3 ± 0.46	*N*/A		-	-	-		-	-	-	
Adamopoulos, 2014	AIMT *n* = 21	8.6 ± 0.5 (C)	8 ± 0.8 (C)	0.05	0.004	-	-	-	-	-	-	-	-	-	-	-	-
	ASIMT *n* = 22	9.1 ± 0.5 (C)	8.9 ± 0.7 (C)	*N’S*		-	-	-		-	-	-		-	-	-	
Hossein Pour, 2019	IMT *n* = 42	-	-	-	-	2.63 ± 0.79	1.38 ± 0.66	<0.001	<0.001	-	-	-	-	-	-	-	-
	S.IMT *n* = 42	-	-	-		2.19 ± 0.89	2.28 ± 0.94	0.036		-	-	-		-	-	-	

*p*-value *: showed intra-group statistical significance. *p*-value **: showed between-groups statistical significance. *N’S* (S) * = non-significant (*p* > 0.05) between groups in CWT at a low velocity; *N’S* (*N*) * = non-significant (*p* > 0.05) between groups in CWT at a usual velocity; *N’S* (F) * = non-significant (*p* > 0.05) between groups in CWT at a rapid speed. * Modified Borg scale was used and varied from 6 to 20. Letters after results showed Borg scale used at different conditions: 6 = after 6 min walking test; C = maximum peak of exercise tolerance; E = performing not specific exercise; F = performing the “corridor walk test” (CWT) at a rapid speed; *n* = CWT at a usual velocity; S = CWT at a low velocity, R = at rest; T = after treadmill test. *N*/A: No data available, or available tables did not permit to determine these values correctly; *N’S*: non-significant (*p* > 0.05); AIMT = AT + IMT; ARIS = combined AT/RT/IMT; AT = Aerobic training; ASIMT: AT + S.IMT; Ctl. = control group; C.IMT: control IMT; H.IMT = high intensity IMT; L.IMT = low intensity IMT; MMRC = Modified Medical Research Council; RT = Resistance training; IMT = Inspiratory muscle training; S.IMT = Sham IMT.

**Table:** (**b**)

Studies	Groups	Fatigue Severity Scale			
		Pre	Post	*p*-Value *	*p*-Value **
Bosnak-Guclu, 2011	IMT *n* = 16	42.73 ± 11.75	29.07 ± 13.96	<0.001	*N’S*
	Sham IMT *n* = 14	42.86 ± 12.67	32.93 ± 15.87	0.008	
Hossein Pour, 2019	IMT *n* = 42	43.86 ± 8.50	28.95 ± 9.11	<0.001	<0.001
	Sham IMT *n* = 42	40.64 ± 10.89	41.47 ± 10.67	0.018	

*p*-value *: showed intra-group statistical significance. *p*-value **: showed between-groups statistical significance. IMT = Inspiratory muscle training; *N’S* = non-significant (*p* > 0.05).

**Table:** (**a**)

Studies	Groups	6 MWT (Feet or Meters) *				CWT (s)				Exercise Period ^1^				NYHA			
		Pre	Post	*p*-Value *	*p*-Value **	Pre	Post	*p*-Value *	*p*-Value **	Pre	Post	*p*-Value *	*p*-Value **	Pre	Post	*p*-Value *	*p*-Value **
Mancini, 1995	T.G. *n* = 8	1110 ± 351 ft	1420 ± 328 ft	<0.001	*N*/A	-	-	-	-	*N*/A	785 ± 230	<0.05	*N*/A	-	-	-	-
	Ctl. *n* = 6	1212 ± 541 ft	1243 ± 565 ft	*N’S*		-	-	-		*N*/A	*N*/A	*N*/A		-	-	-	
Johnson, 1998	IMT *n* = 8	-	-	-	-	93.5 ± 16.4 (S)	−3.8 ± 9.5 (S)	*N*/A	*N’S* (S)	542 ± 383 ″	+152 ± 144 ″	*N*/A	*N’S*	-	-	-	-
						79.8 ± 12.3 (*n*)	−4.4 (*n*)	*N*/A									
						66.1 ± 11.5 (F)	−6.3 (F)	*N*/A									
	C.IMT *n* = 8	-	-	-		96.5 ± 25.7 (S)	−4.1 ± 16.3(S)	*N*/A	*N’S* (*n*)	543 ± 287 ″	+82 ± 118 ″	*N*/A		-	-	-	
						76.1 ± 11.2 (*n*)	+ 1.5 (*n*)	*N*/A	*N’S* (F)								
						66.8 ± 20.2 (F)	−4.1 (F)	*N*/A									
Weiner, 1999	IMT *n* = 10	458 ± 29 ^2^	562 ± 32 ^2^	<0.01	*N*/A	-	-	-	-	-	-	-	-	-	-	-	-
	C.IMT *n* = 6	428 ± 31 ^2^	419 ± 25 ^2^	*N’S*		-	-	-		-	-	-		-	-	-	
Martínez, 2001	IMT *n* = 11	451 ± 78	486 ± 68	<0.05	*N*/A	-	-	-	-	-	-	-	-	-	-	-	-
	C.IMT *n* = 9	430 ± 110	449 ± 102	*N’S*		-	-	-		-	-	-		-	-	-	
Laoutaris, 2004	IMT *n* = 20	367.1 ± 22.3	433.4 ± 16.9	0.000	*N*/A	-	-	-	-	8.7 ± 0.7 ′	9.9 ± 0.7 ′	0.002	*N*/A	-	-	-	-
	C.IMT *n* = 15	343.7 ± 24.8	352.1 ± 22.1	*N’S*		-	-	-		8.2 ± 0.7 ′	7.7 ± 0.6 ′	*N’S*		-	-	-	
Dall’ago, 2006	IMT *n* = 16	449 ± 17	550 ± 17	*N*/A	<0.002	-	-	-	-	298 ± 154 ″	924 ± 503 ″	<0.001	<0.001	-	-	-	-
	S.IMT *n* = 16	432 ± 41	411 ± 60	*N*/A		-	-	-		256 ± 132 ″	246 ± 121 ″	*N’S*		-	-	-	
Laoutaris, 2007	H.IMT *n* = 15	378.2 ± 10.4	404.3 ± 11.9	<0.01	*N’S*	-	-	-	-	9.9 ± 0.5 ′	10.4 ± 0.5 ′	*N’S*	<0.01	-	-	-	-
	L.IMT *n* = 23	358 ± 10	366 ± 16.5	*N’S*		-	-	-		8.3 ± 0.5 ′	8.1 ± 0.5 ′	*N’S*		-	-	-	
Laoutaris, 2008	H.IMT *n* = 14	-	-	-	-	-	-	-	-	9.8 ± 0.5 ′	10.2 ± 0.5 ′	*N’S*	*N’S*	-	-	-	-
	L.IMT *n* = 9	-	-	-		-	-	-		9.1 ± 0.7 ′	9.2 ± 0.6 ′	*N’S*		-	-	-	
Winkelmann, 2009	ATIMT *n* = 12	420 ± 90	500 ± 72	<0.001	*N*/A	-	-	-	-	-	-	-	-	-	-	-	-
	AT *n* = 12	433 ± 108	489 ± 81	<0.05		-	-	-		-	-	-		-	-	-	
Bosnak-Guclu, 2011	IMT *n* = 16	419 ± 123	479 ± 132	<0.001	<0.001	-	-	-	-	-	-	-	-	-	-	-	-
	S.IMT *n* = 14	462 ± 134	476 ± 136	*N’S*		-	-	-		-	-	-		-	-	-	
Laoutaris, 2013	ARIS *n* = 13	-	-	-	-	-	-	-	-	9 ± 2 ′	10.5 ± 1.9 ′	0.001	0.01	2.5 ± 0.5	1.9 ± 0.8	0.001	*N’S*
	AT *n* = 14	-	-	-		-	-	-		9.1 ± 1.2 ′	9.9 ± 0.9 ′	0.04		2.4 ± 0.5	2.2 ± 0.8	*N’S*	
Adamopoulos, 2014	ATIMT *n* = 21	-	-	-	-	-	-	-	-	8.6 ± 2.5 ′	10.1 ± 2.1 ′	<0.001	*N’S*	2.5 ± 0.5	2 ± 0.5	0.001	*N’S*
	ATSIMT *n* = 22	-	-	-		-	-	-		9.8 ± 3.4 ′	10.6 ± 2.8 ′	*N’S*		2.5 ± 0.5	2.1 ± 0.6	0.02	
Palau, 2014	IMT *n* = 14	345 (189–400)	389 (347–423)	<0.001	<0.001	-	-	-	-	-	-	-	-	-	-	-	-
	Ctl. *n* = 12	254 (202–384)	231 (203–375)	*N’S*		-	-	-		-	-	-		-	-	-	
Kawauchi, 2017	MIPRT *n* = 13	393 ± 81	462 ± 69	<0.05	<0.05 ***	-	-	-	-	-	-	-	-	II(5)/III(8)	II(11)/III(1)	0.031	*N*/A ***
	LIPRT *n* = 13	422 ± 114	458 ± 97	<0.05	<0.05 ***	-	-	-	-	-	-	-	-	II(6)/III(7)	II(10)/III(3)	*N’S*	*N*/A ***
	Ctl. *n* = 9	425 ± 47	441 ± 58	*N’S*	-	-	-	-	-	-	-	-	-	II(5)/III(4)	II(5)/III(4)	*N’S*	-
Palau, 2018	IMT *n* = 15	*N*/A	*N*/A	<0.05	*N*/A	-	-	-	-	-	-	-	-	-	-	-	-
	IMTFES *n* = 16	*N*/A	*N*/A	<0.05		-	-	-		-	-	-		-	-	-	
	FES *n* = 15	*N*/A	*N*/A	<0.05		-	-	-		-	-	-		-	-	-	
	Ctl. *n* = 13	*N*/A	*N*/A	*N’S*		-	-	-		-	-	-		-	-	-	
Hossein Pour, 2019	IMT *n* = 42	-	-	-	-	-	-	-	-	-	-	-	-	2.73 ± 0.5	2.1 ± 0.6	0.001	0.003
	S.IMT *n* = 42	-	-	-		-	-	-		-	-	-		2.73 ± 0.8	2.65 ± 0.5	*N’S*	

*p*-value *: showed intra-group statistical significance. *p*-value **: showed between-groups statistical significance. *p*-value ***: p-value of intervention group vs. control at the same time point. * If it is specified, we refer to meters; meanwhile, feet were specified by the abbreviation ft. ^1^: Expressed as minutes (′) and seconds (″). ^2^: A variation of 6 MWT was used and using the 12 MWT; *N* = usual velocity in the CWT. S = slow velocity in the CWT; F = rapid velocity in the CWT; *N*/A: no data available or not exactly determined due to these values are shown in graphs; *N’S*: non-significant (*p* > 0.05). ARIS = combined AT/RT/IMT; AT = Aerobic training; ATIMT = AT + IMT; ATSIMT: AT + S.IMT; Ctl. = control group; C.IMT = control IMT; CWT = “corridor walk test”; FES = Functional electrical stimulation; H.IMT = high intensity IMT; IMT = Inspiratory muscle training; IMTFES = IMT + FES; L.IMT = low intensity IMT; MTW = minutes walking test; RT = Resistance training; S.IMT: sham IMT; T.G = training group.

**Table:** (**b**)

Studies	Groups	Hand Grip Strength				Quadriceps Strength			
		Pre	Post	*p* *	*p* **	Pre	Post	*p* *	*p* **
Bosnak-Guclu, 2011	IMT *n* = 16	-	-	-	-	241 ± 106 *n*	302 ± 112 *n*	<0.001	0.031
	S.IMT *n* = 14	-	-	-		292 ± 103 *n*	309 ± 133 *n*	*N’S*	
Laoutaris, 2013	ARIS *n* = 13	-	-	-	-	1.9 ± 0.3 *N*·m	2.4 ± 0.38 *N*·m	<0.001	0.003
	AT *n* = 14	-	-	-		1.8 ± 0.1 *N*·m	1.9 ± 0.2 *N*·m	*N’S*	
Marco 2013	IMT *n* = 11	26.9 ± 10.4 *N* (D)	29.8 ± 10.9 *N* (D)	*N*/A	*N’S* (D)	-	-	-	-
		26.6 ± 11.4 *N* (NO)	27.3 ± 8.1 *N* (NO)						
	S.IMT *n* = 11	31.3 ± 9.9 *N* (D)	31.2 ± 11.1 *N* (D)	*N*/A	*N’S* (NO)	-	-	-	
		30.4 ± 9.5 *N* (NO)	30.5 ± 10.2 *N* (NO)						
Kawauchi, 2017	MIRPT *n* = 13	*N*/A	*N*/A	-	-	234 ± 75 *N*	279 ± 79 *N*	<0.05	<0.05 ***
	LIRPT *n* = 13	*N*/A	*N*/A	-	-	248 ± 80 *N*	290 ± 94 *N*	<0.05	<0.05 ***
	Ctl. *n* = 9	*N*/A	*N*/A	-	-	204 ± 45 *N*	203 ± 54 *N*	*N’S*	-
Hornikx, 2019	RHIIT *n* = 10	-	-	-	-	107 ± 32 *N*·m	+19.3 ± 11.8 *N*·m	<0.01	<0.01
	SP *n* = 10	-	-	-		144 ± 52 *N*·m	−6.89 ± 19 *N*·m	*N’S*	
Taya, 2019	ATIMT *n* = 1	29.8 kg	29.2 kg	*N*/A	-	164 *N* (D) 154 *N* (NO)	223 *N* (D) 185 *N* (NO)	*N*/A	-

*p* *: showed intra-group statistical significance. *p* **: showed between-groups statistical significance. *p*-value ***: *p*-value of intervention group vs. control at the same time point. ARIS = combined AT/RT/IMT; AT = Aerobic training; ATIMT = AT + IMT; Ctl. = control group; D = Dominant hand; IMT = Inspiratory muscle training; LIPRT = Low intensity (IMT) plus resistance training; M = Men; MIPRT = Moderate intensity (IMT) plus resistance training; *N*/A = No data available; NO = Non-dominant hand; RPE = Rate of perceived exertion; RT = Resistance training; RHIIT = Resistance training supplemented HIIT; S.IMT = sham IMT.

**Table 5:** Parameter related to VO_2._

Studies	Groups	Peak VO_2_ (mL/kg/min)				VO_2 AT_ (mL/kg/min)				CP (mmHg·mL/kg/min)				OUES (mL·min^−1^O_2_/Lmin^−1^VE)			
		Pre	Post	*p* *	*p* **	Pre	Post	*p* *	*p* **	Pre	Post	*p* *	*p* **	Pre	Post	*p* *	*p* **
Mancini, 1995	T.G. *n* = 8	11.4 ± 3.3	13.3 ± 2.7	<0.05	*N*/A	*N*/A	7.8 ± 1.3	*N*/A	*N*/A	-	-	-	-	-	-	-	-
	Ctl. *n* = 6	16.1 ± 5.5	15 ± 6	*N’S*		*N*/A	*N*/A	*N*/A		-	-	-		-	-	-	
Weiner, 1999	IMT *n* = 10	13.1 ± 0.8	*N*/A	*N’S*	*N*/A	-	-	-	-	-	-	-	-	-	-	-	-
	C.IMT *n* = 6	13.5 ± 0.9	*N*/A	*N’S*		-	-	-		-	-	-		-	-	-	
Martínez, 2001	IMT *n* = 11	19 ± 3	21.6 ± 5	<0.05	*N*/A	-	-	-	-	-	-	-	-	-	-	-	-
	C.IMT *n* = 9	16 ± 5	18.6 ± 7	<0.05		-	-	-		-	-	-		-	-	-	
Laoutaris, 2004	IMT *n* = 20	15.4 ± 0.9	17.8 ± 1.2	0.002	*N*/A	13.1 ± 1	13.4 ± 1	*N’S*	*N*/A	-	-	-	-	-	-	-	-
	C.IMT *n* = 15	14.7 ± 1	14.7 ± 1	*N’S*		12.2 ± 1.1	11.3 ± 1	*N’S*		-	-	-		-	-	-	
Dall’ago, 2006	IMT *n* = 16	17 ± 0.6	21 ± 0.7	<0.001	<0.001	-	-	-	-	2829 ± 409	3696 ± 524	<0.001	<0.001	-	-	-	-
	S.IMT *n* = 16	17 ± 0.6	17 ± 0.8	*N’S*		-	-	-		2714 ± 505	2592 ± 421	*N’S*		-	-	-	
Laoutaris, 2007	H.IMT *n* = 15	17.3 ± 0.9	19.4 ± 1.2	<0.01	<0.01	14.3 ± 1.1	14.8 ± 1.2	*N’S*	*N’S*	-	-	-	-	-	-	-	-
	L.IMT *n* = 23	15.7 ± 0.8	14.8 ± 0.8	*N’S*		13.1 ± 0.9	11.5 ± 0.8	*N’S*		-	-	-		-	-	-	
Laoutaris, 2008	H.IMT *n* = 14	17.1 ± 0.7	19 ± 1.2	0.01	*N’S*	14.1 ± 1.1	14.4 ± 1.2	*N’S*	*N’S*	1908 ± 97	2343 ± 169	0.002	*N’S*	-	-	-	-
	L.IMT *n* = 9	17.7 ± 1.3	17.3 ± 1.5	*N’S*		14.8 ± 1.3	12.9 ± 1.4	*N’S*		2192 ± 232	2127 ± 225	*N’S*		-	-	-	
Stein, 2009	IMT *n* = 16	-	-	-	-	-	-	-	-	-	-	-	-	1554 ± 617	2037 ± 747	<0.01	<0.01
	S.IMT *n* = 16	-	-	-		-	-	-		-	-	-		1428 ± 626	1597 ± 615	*N’S*	
Winkelmann, 2009	ATIMT *n* = 12	15.1 ± 4.2	19.7 ± 4.1	<0.001	<0.001	-	-	-	-	2250 ± 815	3276 ± 857	<0.001	<0.001	1323 ± 766	2040 ± 545	<0.001	<0.1
	AT *n* = 12	16.1 ± 4.6	19.2 ± 4.2	<0.001		-	-	-		2569 ± 880	3065 ± 869	*N’S*		1398 ± 657	1880 ± 617	*N’S*	
Mello, 2012	IMT *n* = 15	14.4 ± 0.7	18.9 ± 0.8	0.002	<0.05	-	-	-	-	-	-	-	-	-	-	-	-
	Ctl. *n* = 12	16.2 ± 0.5	16.3 ± 0.6	*N’S*		-	-	-		-	-	-		-	-	-	
Laoutaris, 2013	ARIS *n* = 13	16.8 ± 5.2	19.6 ± 6.2	0.01	*N’S*	-	-	-	-	2337 ± 340	3089 ± 984	0.001	0.05	-	-	-	-
	AT *n* = 14	17.6 ± 3.6	19.5 ± 4.1	0.04		-	-	-		2527 ± 149	2697 ± 274	0.03		-	-	-	
Adamopoulos, 2014	ATIMT *n* = 21	17.3 ± 5.6	18.9 ± 5.3	0.008	*N’S*	-	-	-	-	2583 ± 1092	2799 ± 1051	*N’S*	*N’S*	-	-	-	-
	ATSIMT *n* = 22	18.6 ± 4.4	20.2 ± 5.5	0.04		-	-	-		2859 ± 901	3079 ± 1039	*N’S*		-	-	-	
Palau, 2014	IMT *n* = 14	10.3 (7.7–12.8)	13.2 (10.6–14.6)	<0.001	<0.001	8.4 (6.6–10)	10.2 (9–11.6)	<0.001	0.001	-	-	-	-	-	-	-	-
	Ctl. *n* = 12	10 (6.8–10.9)	9 (6.9–10.5)	*N’S*		8 (6.3–9.3)	7.4 (6.3–8)	*N’S*		-	-	-		-	-	-	
Palau, 2018	IMT *n* = 15	9.9 ± 2.3	12.6 ± 3.4	<0.001	*N*/A	-	-	-	-	-	-	-	-	-	-	-	-
	IMTFES *n* = 16	10.7 ± 2.9	12.9 ± 3.7	<0.001		-	-	-		-	-	-		-	-	-	
	FES *n* = 15	9.6 ± 2.0	11.8 ± 2.6	<0.001		-	-	-		-	-	-		-	-	-	
	Ctl. *n* = 13	9.3 ± 2.5	8.8 ± 2.6	*N’S*		-	-	-		-	-	-		-	-	-	
Palau 2019	IMT *n* = 45	10.4 ± 2.8	12.6 ± 3.2	<0.001	-	-	-	-	-	-	-	-	-	-	-	-	-
Hornikx 2019	RHIIT *n* = 10	13.5 ± 3.7	3.4 ± 2.6	<0.01	*N’S*	-	-	-	-	-	-	-	-	-	-	-	-
	SP *n* = 10	14.7 ± 3.6	3.3 ± 1.8	<0.01		-	-	-		-	-	-		-	-	-	
Taya, 2019	AT + IMT *n* = 1	6.8	10.9	*N*/A	-	-	-	-	-	-	-	-	-	-	-	-	-

*p* *: showed intra-group statistical significance. *p* **: showed between-groups statistical significance. ARIS = combined AT/RT/IMT; AT = Aerobic training; ATIMT = AT + IMT; ATSIMT: AT + S.IMT; Ctl. = control group; C.IMT = control IMT; CP = Circulatory power; FES = Functional electrical stimulation; HIIT = high intensity interval training; H.IMT = high intensity IMT; L.IMT = low intensity IMT; IMT = Inspiratory muscle training; IMTFES = IMT + FES; *N*/A = No data available; *N’S* = non-significant (*p* > 0.05); OUES = Oxygen absorption efficiency slope; RHIIT = Resistance training supplemented HIIT; RT = Resistance training; S.IMT = Sham IMT; SP = Standard protocol; VO_2 AT_ = VO_2_ anaerobic threshold.

**Table 6:** Ventilation per minute (VE)/VCO_2_ and VE.

Studies	Groups	VE/VCO_2_ Slope				VE (l/min)			
		Pre	Post	*p*-Value *	*p*-Value **	Pre	Post	*p*-Value *	*p*-Value **
Mancini, 1995	Training *n* = 8	38.2 ± 5.9	38.3 ± 5.3	*N’S*	*N*/A	44 ± 15	55 ± 12	<0.05	*N*/A
	Ctl. *n* = 6	*N*/A	*N*/A	*N*/A		*N*/A	*N*/A	*N*/A	
Martínez, 2001	IMT *n* = 11	*N*/A	*N*/A	*N’S*	*N*/A	-	-	-	-
	C.IMT *n* = 9	*N*/A	*N*/A	*N’S*		-	-	-	
Laoutaris, 2004	IMT *n* = 20	36.7 ± 1.6	36.2 ± 2.1	*N’S*	*N*/A	51.9 ± 3.9	63.6 ± 5.8	0.003	*N*/A
	C.IMT *n* = 15	40.5 ± 1	42.5 ± 2.3	*N’S*		54.5 ± 4.2	51.7 ± 3.2	*N’S*	
Dall’ago 2006	IMT *n* = 16	35 ± 3.5	30 ± 3	<0.001	<0.001	48 ± 2.7	62 ± 4	<0.001	<0.001
	S.IMT *n* = 16	37 ± 4	37 ± 4	*N’S*		47 ± 3	49 ± 4	*N’S*	
Laoutaris, 2007	H.IMT *n* = 15	33.9 ± 2.2	33.4 ± 2	*N’S*	<0.05	56.9 ± 4.7	63.5 ± 6.6	*N’S*	*N’S*
	LIMT *n* = 23	38.9 ± 1.5	40.4 ± 1.8	*N’S*		57.3 ± 3.4	55.3 ± 2.8	*N’S*	
Laoutaris, 2008	H.IMT *n* = 14	34.2 ± 2.1	33.9 ± 2.1	*N’S*	*N’S*	57.2 ± 5.1	64.1 ± 7.1	*N’S*	*N’S*
	L.IMT *n* = 9	35.3 ± 2.3	35.2 ± 2.4	*N’S*		61.3 ± 5.2	61 ± 4.2	*N’S*	
Winkelmann, 2009	ATIMT *n* = 12	44 ± 5	30 ± 7	<0.001	<0.001	48 ± 21	56 ± 30	<0.001	*N’S*
	AT *n* = 12	37 ± 7	33 ± 6	*N’S*		48 ± 17	52 ± 13	*N’S*	
Mello, 2012	IMT *n* = 15	37.3 ± 1.1	31.3 ± 1.1	0.001	<0.05	-	-	-	-
	Ctl. *n* = 12	36.6 ± 1.2	38.7 ± 3	*N’S*		-	-	-	
Laoutaris, 2013	ARIS *n* = 13	37.9 ± 6.4	35.8 ± 5.8	0.009	*N’S*	66.9 ± 39	70 ± 29	*N’S*	*N’S*
	AT *n* = 14	35.9 ± 4.8	34.8 ± 5.4	*N’S*		54.1 ± 13	58.4 ± 12	*N’S*	
Adamopoulos, 2014	ATIMT *n* = 21	36.4 ± 5.6	35.8 ± 6.6	*N’S*	*N’S*	63.3 ± 20.3	73.6 ± 17.3	0.002	*N’S*
	ATSIMT *n* = 22	37.5 ± 6.9	36.2 ± 6.4	*N’S*		64.2 ± 15.6	65.3 ± 14.1	*N’S*	
Palau, 2014	IMT *n* = 14	31 (23–35.1)	26 (22–30)	0.016	0.007	-	-	-	-
	Ctl. *n* = 12	33.8 (26.5–40)	34.9 (30–41)	*N’S*		-	-	-	
Palau, 2018	IMT *n* = 15	38.5 ± 5.7	35.3 ± 5.8	0.011	*N*/A	-	-	-	-
	IMT + FES *n* = 16	38.4 ± 4.5	35.4 ± 4.5	0.009		-	-	-	
	FES *n* = 15	35.1 ± 4.5	34.5 ± 6.3	*N’S*		-	-	-	-
	Ctl. *n* = 13	37.9 ± 6.3	37 ± 5.9	*N’S*		-	-	-	
Hornikx, 2019	RHIIT *n* = 10	39 ± 12	−4.4 ± 12	*N’S*	*N’S*	-	-	-	-
	SP *n* = 10	34 ± 7	+1.3 ± 9.8	*N’S*		-	-	-	
Taya, 2019	AT + IMT *n* = 1	57.5	45.3	*N*/A	-	-	-	-	-

*p*-value *: showed intra-group statistical significance. *p*-value **: showed between-groups statistical significance. ARIS = combined AT/RT/IMT; AT = Aerobic training; ATIMT = AT + IMT; ATSIMT = AT + S.IMT; Ctl. = control group; C.IMT = control IMT; FES = Functional electrical stimulation; HIIT = high intensity interval training; H.IMT = high intensity IMT; L.IMT = low intensity IMT; IMT = Inspiratory muscle training; N/A = No data available; *N’S* = non-significant (*p* > 0.05); RHIIT = Resistance training supplemented HIIT; RT = Resistance training; S.IMT = sham IMT; SP = Standard protocol; VE = Ventilation per minute.

**Table 7:** Cardiovascular parameters.

Studies	Groups	HR at Rest (bpm)				Maximum HR (bpm)				LVEF (%)				LVEDD (mm)			
		Pre	Post	*p* *	*p* **	Pre	Post	*p* *	*p* **	Pre	Post	*p* *	*p* **	Pre	Post	*p* *	*p* **
Laoutaris, 2004	IMT *n* = 20	80.2 ± 3	76.8 ± 3.3	0.04	*N*/A	134.9 ± 5.5	132.3 ± 5.9	*N’S*	*N*/A	23.5 ± 1.5	24.5 ± 1.5	*N’S*	*N*/A	70.94 ± 2.1	70.89 ± 2	*N’S*	*N*/A
	C.IMT *n* = 15	78.6 ± 5.3	76.1 ± 4.7	*N’S*		129.7 ± 6.5	129.3 ± 8.6	*N’S*		25.7 ± 2.1	25.3 ± 2.2	*N’S*		67.47 ± 2.9	68.13 ± 2.9	*N’S*	
Laoutaris, 2007	H.IMT *n* = 15	82 ± 5	81 ± 3	*N’S*	*N’S*	140 ± 7	134 ± 6	<0.05	*N’S*	-	-	-	-	-	-	-	-
	L.IMT *n* = 23	82 ± 4	81 ± 4	*N’S*		133 ± 5	132 ± 6	*N’S*		-	-	-		-	-	-	
Laoutaris, 2008	H.IMT *n* = 14	83 ± 6	80 ± 3	*N’S*	*N’S*	140 ± 8	134 ± 7	0.03	*N’S*	-	-	-	-	-	-	-	-
	L.IMT *n* = 9	86 ± 5	89 ± 5	*N’S*		140 ± 7	138 ± 8	*N’S*		-	-	-		-	-	-	
Winkelmann, 2009	ATIMT *n* = 12	-	-	-	-	136 ± 24	135 ± 33	*N’S*	*N’S*	-	-	-	-	-	-	-	-
	AT *n* = 12	-	-	-		144 ± 26	142 ± 24	*N’S*		-	-	-		-	-	-	
Mello, 2012	IMT *n* = 15	70.3 ± 3.3	68.5 ± 3.4	*N’S*	*N*/A	-	-	-	-	-	-	-	-	-	-	-	-
	Ctl. *n* = 12	65.6 ± 3	63.6 ± 2.3	*N’S*		-	-	-		-	-	-		-	-	-	
Laoutaris, 2013	ARIS *n* = 13	76 ± 16	71 ± 18	*N’S*	*N’S*	130 ± 26	134 ± 21	*N’S*	*N’S*	27.8 ± 8	30.4 ± 8.2	0.003	*N’S*	69.4 ± 4.6	67.5 ± 3.9	0.01	*N’S*
	AT *n* = 14	81 ± 12	78 ± 12	*N’S*		140 ± 18	141 ± 17	*N’S*		30.6 ± 5.4	33.4 ± 5.7	0.01		66.1 ± 3.8	65.3 ± 3.7	*N’S*	
Adamopoulos, 2014	ATIMT *n* = 21	74 ± 11	76 ± 12	*N’S*	*N’S*	124 ± 21	129 ± 24	*N’S*	*N’S*	28 ± 7	36 ± 11	0.005	*N’S*	65 ± 9	64 ± 9	*N’S*	*N’S*
	ASIMT *n* = 22	78 ± 15	76 ± 11	*N’S*		140 ± 26	138 ± 23	*N’S*		30 ± 5	36 ± 9	0.002		63 ± 7	62 ± 6	*N’S*	
Palau, 2014	IMT *n* = 14	72 (63–82)	67 (54–71)	<0.001	0.01	121 (102–134)	124 (104–138)	0.004	0.004	69 (63–77)	68 (60–72)	*N’S*	*N’S*	-	-	-	-
	Ctl. *n* = 12	69 (61–90)	70 (63–82)	*N’S*		113 (110–125)	111 (98–119)	<0.001		76 (68–83)	78 (69–81)	*N’S*		-	-	-	
Moreno, 2017	IMT *n* = 15	70 ± 12	63 ± 5.8	<0.05	*N*/A	-	-	-	-	-	-	-	-	-	-	-	-
	Ctl. *n* = 13	69 ± 16.5	68 ± 9.8	*N’S*		-	-	-		-	-	-		-	-	-	

*p* *: showed intra-group statistical significance. *p* **: showed between-groups statistical significance. ARIS = combined AT/RT/IMT; AT = Aerobic training; ATIMT = AT + IMT; ASIMT = AT + S.IMT; Bpm = Beats per minute.; Ctl. = control group; C.IMT = control IMT; H.IMT = high intensity IMT; HR = Heart rate; L.IMT = low intensity IMT; LVEDD = Diameter of the left ventricle at the end of diastole; LVEF = left ventricular ejection fraction; IMT = Inspiratory muscle training; *N*/A = No data available; *N’S* = non-significant (*p* > 0.05); RT = Resistance training; S.IMT = sham IMT.

**Table 8:** Biomarkers.

Studies	Groups	NT-proBNP (pg/mL)				CRP (ng/l)				CA-125 (U/mL)			
		Pre	Post	*p*-Value *	*p*-Value **	Pre	Post	*p*-Value *	*p*-Value **	Pre	Post	*p*-Value *	*p*-Value **
Laoutaris, 2007	H.IMT *n* = 15	-	-	-	-	7.3 ± 2.6	8.5 ± 2.7	*N’S*	*N’S*	-	-	-	-
	L.IMT *n* = 23	-	-	-		7.1 ± 1.9	8.1 ± 1.8	*N’S*		-	-	-	
Laoutaris, 2008	H.IMT *n* = 14	527 ± 74	530 ± 78	*N’S*	*N’S*	-	-	-	-	-	-	-	-
	L.IMT *n* = 9	675 ± 126	637 ± 121	*N’S*		-	-	-		-	-	-	
Marco, 2013	H.IMT *n* = 11	1677 (SD 1658)	1593 (SD 1308)	*N*/A	*N’S*	0.6 (SD 0.6)	0.4 (SD 0.4)	*N*/A	*N’S*	-	-	-	-
	S.IMT *n* = 11	2212 (SD 3155)	2294 (SD 3567)	*N*/A		1.5 (SD 1.5)	3.3 (SD 3.9)	*N*/A		-	-	-	
Adamopoulos, 2014	ATIMT *n* = 21	1046 ± 766	790 ± 683	*N’S*	0.004	2.8 ± 1.5	1.4 ± 0.8	0.05	0.03	-	-	-	-
	ATSIMT *n* = 22	1525 ± 1657	1866 ± 1196	*N’S*		4.6 ± 5.8	3.5 ± 2.9	*N’S*		-	-	-	
Palau, 2014	IMT *n* = 14	983 (325–1932)	674 (127–1878)	*N’S*	*N’S*	-	-	-	-	13 (8–29)	12 (7–23)	*N’S*	*N’S*
	Ctl. *n* = 12	1314 (255–1868)	1525 (204–2799)	*N’S*		-	-	-		16 (11–36)	22 (14–37)	*N’S*	
Palau, 2018	IMT *n* = 15	1316 (282–3546)	910 (183–2301)	*N’S*	*N*/A	-	-	-	-	15 (9–49)	13 (8–19)	*N’S*	*N*/A
	IMT + FES *n* = 16	767 (369–1974)	615 (344–1242)	*N’S*		-	-	-		18 (10–23)	17 (10–21)	*N’S*	
	FES *n* = 15	567 (302–1583)	667 (247–1310)	*N’S*		-	-	-		15 (8–19)	14 (10–16)	*N’S*	
	Ctl. *n* = 13	755 (383–999	983 (246–1193)	*N’S*		-	-	-		11 (9–18)	16 (8–21)	*N’S*	

*p*-value *: showed intra-group statistical significance. *p*-value **: showed between-groups statistical significance. AT = aerobic training; ATIMT = AT + IMT; ATSIMT = AT + S.IMT; CA-125 = 125 serum carbohydrate antigen; CRP = C-reactive protein; Ctl. = control group; FES = Functional electrical stimulation; H.IMT = high intensity IMT; IMT = Inspiratory muscle training; L.IMT = low intensity IMT; *N*/A: no data available; *N’S* = non-significant (*p* > 0.05); NT-proBNP = *N*-terminal pro-brain natriuretic peptide; S.IMT = sham IMT.

**Table 9:** Quality of life.

Studies	Groups	MLwHFQ				SF-36				CHFJ			
		Pre	Post	*p*-Value *	*p*-Value **	Pre	Pro	*p*-Value *	*p*-Value **	Pre	Post	*p*-Value *	*p*-Value **
Johnson, 1998	IMT *n* = 8	-	-	-	-	-	-	-	-	5.3 ± 0.9	+0.55 ± 0.48	*N*/A	*N’S*
	C.IMT *n* = 8	-	-	-		-	-	-		4.6 ± 0.8	+0.06 ± 0.38	*N*/A	
Laoutaris, 2004	IMT *n* = 20	25.2 ± 4	21.1 ± 3.5	0.004	*N*/A	-	-	-	-	-	-	-	-
	C.IMT *n* = 15	22.9 ± 2.6	22.6 ± 2.5	*N’S*		-	-	-		-	-	-	
Dall’ago, 2006	IMT *n* = 16	27 ± 4	6 ± 2	*N*/A	<0.001	-	-	-	-	-	-	-	-
	S.IMT *n* = 16	30 ± 13	30 ± 13	*N*/A		-	-	-		-	-	-	
Padula, 2009	IMT *n* = 15	-	-	-	-	29.15	*N*/A	*N’S*	*N’S*	-	-	-	-
	C.IMT *n* = 17	-	-	-		29.11	*N*/A	*N’S*		-	-	-	
Winkelmann, 2009	ATIMT *n* = 12	45 ± 21	20 ± 15	<0.001	*N*/A	-	-	-	-	-	-	-	-
	AT *n* = 12	45 ± 18	18 ± 15	<0.05		-	-	-		-	-	-	
Bosnak-Guclu, 2011	IMT *n* = 16	-	-	-	-	46 ± 28 (*p*)	67 ± 24 (*p*)	<0.001	*N’S* (*p*)	-	-	-	-
						58 ± 24 (M)	70 ± 21 (M)	0.004					
	S.IMT *n* = 14	-	-	-		52 ± 23 (*p*)	69 ± 22 (*p*)	<0.001	*N’S* (M)	-	-	-	
						55 ± 24 (M)	72 ± 22 (M)	0.001					
Mello, 2012	IMT *n* = 15	26.6 ± 3.8	9.2 ± 2.4	<0.05	<0.05	-	-	-	-	-	-	-	-
	Ctl. *n* = 12	30.8 ± 6.1	32.7 ± 5.6	*N’S*		-	-	-		-	-	-	
Laoutaris, 2013	ARIS *n* = 13	41.6 ± 3.6	33.7 ± 3.2	<0.001	0.03	-	-	-	-	-	-	-	-
	AT *n* = 14	42.4 ± 4.8	37.8 ± 7	*N’S*		-	-	-		-	-	-	
Marco, 2013	IMT *n* = 11	-	-	-	-	*N*/A	*N*/A	*N*/A	*N*/A	-	-	-	-
	S.IMT *n* = 11	-	-	-		*N*/A	*N*/A	*N*/A		-	-	-	
Adamopoulos, 2014	AT + IMT *n* = 21	38 ± 10.4	27.7 ± 11.3	<0.001	0.002	-	-	-	-	-	-	-	-
	AT + S.IMT *n* = 22	42 ± 8.1	38.8 ± 8.4	*N’S*		-	-	-		-	-	-	
Palau, 2014	IMT *n* = 14	41 (34–48)	30 (25–35)	0.002	0.037	-	-	-	-	-	-	-	-
	Ctl. *n* = 12	48 (25–61)	45 (24–52)	*N’S*		-	-	-		-	-	-	
Kawauchi, 2017	MIRPT *n* = 13	36 ± 23	20 ± 10	<0.05	*N’S* ***	-	-	-	-	-	-	-	-
	LIRPT *n* = 13	42 ± 24	28 ± 19	<0.05	*N’S* ***	-	-	-	-	-	-	-	-
	Ctl. *n* = 9	37 ± 25	28 ± 21	<0.05	-	-	-	-	-	-	-	-	-
Moreno, 2017	IMT *n* = 15	*N*/A	*N*/A	<0.001	<0.01	-	-	-	-	-	-	-	-
	Ctl. *n* = 13	*N*/A	*N*/A	*N’S*		-	-	-		-	-	-	
Palau, 2018	IMT *n* = 15	42.3 ± 16.5	27.2 ± 14.5	<0.001	*n*/A	-	-	-	-	-	-	-	-
	IMT + FES *n* = 16	34.9 ± 21.6	25.3 ± 14.1	<0.001		-	-	-		-	-	-	
	FES *n* = 15	39.7 ± 21.2	31.1 ± 20.5	0.014		-	-	-		-	-	-	
	Ctl. *n* = 13	42.8 ± 21.3	40.4 ± 22.4	*N’S*		-	-	-		-	-	-	
Hornikx, 2019	RHIIT *n* = 10	33 ± 17	−18.2 ± 13.1	<0.01	*N’S*	-	-	-	-	-	-	-	-
	SP *n* = 10	24 ± 20	−10.4 ± 19.5	*N’S*		-	-	-		-	-	-	

*p*-value *: showed intra-group statistical significance. *p*-value **: showed between-groups statistical significance. *p*-value ***: *p*-value of intervention group vs. control at the same time point. ARIS = combined AT/RT/IMT; AT = Aerobic training; ATIMT = AT + IMT; Ctl. = control group; C.IMT = control IMT; FES = Functional electrical stimulation; HIIT = high intensity interval training; IMT = Inspiratory muscle training; LIPRT = Low intensity (IMT) plus resistance training; M = mental health; MLwHFQ = Minnesota Living with Heart Failure Questionnaire; MIPRT = Moderate intensity (IMT) plus resistance training; *N*/A = No data available; *N’S* = non-significant (*p* > 0.05); *p* = physical health; RHIIT = Resistance training supplemented HIIT; SF-36 = Short-Form 36 Questionnaire; S.IMT = sham IMT; SP = Standard protocol; RT = Resistance training.

## Supplementary Materials

The following are available online at https://www.mdpi.com/2077-0383/9/6/1710/s1. Table S1: Excluded articles and causes of exclusion.
