# Supplementary material for: Inspiratory Muscle Training in Patients with Heart Failure
Source: J Clin Med. 2020 Jun 2;9(6):1710. doi: 10.3390/jcm9061710 (PMC7356942; doi:10.3390/jcm9061710)
Supplement: Supplementary file 1 [file jcm-09-01710-s001.pdf]

Supplemental file 1. Excluded articles and causes of exclusion.

Articles	Causes of exclusion
Cahalin LP, Arena RA. Breathing exercises and inspiratory muscle training in heart failure. Heart Fail Clin. 2015;11(1):149-172. doi:10.1016/j.hfc.2014.09.002	Narrative review.
Wu J, Kuang L, Fu L. Effects of inspiratory muscle training in chronic heart failure patients: A systematic review and meta-analysis. Congenit Heart Dis. 2018;13(2):194-202. doi:10.1111/chd.12586	Systematic Review and Meta-Analysis.
Göhl O, Walker DJ, Walterspacher S, et al. Atemmuskeltraining: State-of-the-Art [Respiratory Muscle Training: State of the Art]. Pneumologie. 2016;70(1):37-48. doi:10.1055/s-0041-109312	Non-experimental studies.
Hirai DM, Musch TI, Poole DC. Exercise training in chronic heart failure: improving skeletal muscle O2 transport and utilization. Am J Physiol Heart Circ Physiol. 2015;309(9):H1419-H1439. doi:10.1152/ajpheart.00469.2015	Non-experimental studies.
Wang MH, Yeh ML. Respiratory training interventions improve health status of heart failure patients: A systematic review and network meta-analysis of randomized controlled trials. World J Clin Cases. 2019;7(18):2760-2775. doi:10.12998/wjcc.v7.i18.2760	Systematic Review and Meta-Analysis.
Lopes CP, Danzmann LC, Moraes RS, et al. Yoga and breathing technique training in patients with heart failure and preserved ejection fraction: study protocol for a randomized clinical trial. Trials. 2018;19(1):405. Published 2018 Jul 28. doi:10.1186/s13063-018-2802-5	Not used IMT.
Giallauria F, Piccioli L, Vitale G, Sarullo FM. Exercise training in patients with chronic heart failure: A new challenge for Cardiac Rehabilitation Community. Monaldi Arch Chest Dis. 2018;88(3):987. Published 2018 Sep 6. doi:10.4081/monaldi.2018.987	Review.
Neto MG, Martinez BP, Conceição CS, Silva PE, Carvalho VO. Combined Exercise and Inspiratory Muscle Training in Patients With Heart Failure: A SYSTEMATIC REVIEW AND META-ANALYSIS. J Cardiopulm Rehabil Prev. 2016;36(6):395-401. doi:10.1097/HCR.0000000000000184	Systematic Review and Meta-Analysis.
Sadek Z, Salami A, Joumaa WH, Awada C, Ahmaidi S, Ramadan W. Best mode of inspiratory muscle training in heart failure patients: a systematic review and meta-analysis. Eur J Prev Cardiol. 2018;25(16):1691-1701. doi:10.1177/2047487318792315	Systematic Review and Meta-Analysis.
Gomes Neto M, Ferrari F, Helal L, Lopes AA, Carvalho VO, Stein R. The impact of high-intensity inspiratory muscle training on exercise capacity and inspiratory muscle strength in heart failure with reduced ejection fraction: a systematic review and meta-analysis. Clin Rehabil. 2018;32(11):1482-1492. doi:10.1177/0269215518784345	Systematic Review and Meta-Analysis.
Bjarnason-Wehrens B, Predel HG. Inspiratory muscle training - an inspiration for more effective cardiac rehabilitation in heart failure patients?. Eur J Prev Cardiol. 2018;25(16):1687-1690. doi:10.1177/2047487318798917	Review.
de Abreu RM, Rehder-Santos P, Minatel V, Dos Santos GL, Catai AM. Effects of inspiratory muscle training on cardiovascular autonomic control: A systematic review. Auton Neurosci. 2017;208:29-35. doi:10.1016/j.autneu.2017.09.002	Systematic review.
Wong E, Selig S, Hare DL. Respiratory muscle dysfunction and training in chronic heart failure. Heart Lung Circ. 2011;20(5):289-294. doi:10.1016/j.hlc.2011.01.009	Review.
Silva IS, Fregonezi GA, Dias FA, Ribeiro CT, Guerra RO, Ferreira GM. Inspiratory muscle training for asthma. Cochrane Database Syst Rev. 2013;2013(9):CD003792. Published 2013 Sep 8. doi:10.1002/14651858.CD003792.pub2	Other conditions in addition to HF such as asthma.
Plentz RD, Sbruzzi G, Ribeiro RA, Ferreira JB, Dal Lago P. Inspiratory muscle training in patients with heart failure: meta-analysis of randomized trials. Arq Bras Cardiol. 2012;99(2):762-771. doi:10.1590/s0066-782x2012001100011	Meta-Analysis.
Lage SM, Britto RR, Brandão DC, Pereira DAG, Andrade AD, Parreira VF. Can diaphragmatic breathing modify chest wall volumes during inspiratory loaded breathing in patients with heart failure?. Braz J Phys Ther.	Not used IMT.

2018;22(6):452-458. doi:10.1016/j.bjpt.2018.04.005	
Nakagawa NK, Diz MA, Kawauchi TS, et al. Risk Factors for Inspiratory Muscle Weakness in Chronic Heart Failure. Respir Care. 2020;65(4):507-516. doi:10.4187/respcare.06766	Non-experimental studies.
Ribeiro JP, Chiappa GR, Neder JA, Frankenstein L. Respiratory muscle function and exercise intolerance in heart failure. Curr Heart Fail Rep. 2009;6(2):95-101. doi:10.1007/s11897-009-0015-7	Non-experimental studies.
Cahalin LP, Arena R, Guazzi M, et al. Inspiratory muscle training in heart disease and heart failure: a review of the literature with a focus on method of training and outcomes [published correction appears in Expert Rev Cardiovasc Ther. 2013 Apr;11(4):520]. Expert Rev Cardiovasc Ther. 2013;11(2):161-177. doi:10.1586/erc.12.191	Narrative review.
Laohachai K, Winlaw D, Selvadurai H, et al. Inspiratory Muscle Training Is Associated With Improved Inspiratory Muscle Strength, Resting Cardiac Output, and the Ventilatory Efficiency of Exercise in Patients With a Fontan Circulation. J Am Heart Assoc. 2017;6(8):e005750. Published 2017 Aug 21. doi:10.1161/JAHA.117.005750	Other conditions in addition to HF such as “Fontan circulation”.
Zeren M, Demir R, Yigit Z, Gurses HN. Effects of inspiratory muscle training in patients with atrial fibrillation. European heart journal. 2015	Other conditions in addition to HF such as atrial fibrillation.
NCT01410279 . Inspiratory Muscle Training in Pulmonary Hypertension. https://clinicaltrials.gov/show/NCT01410279 2011	Other conditions in addition to HF such as pulmonary hypertension.
Sbruzzi G, Dal Lago P, Ribeiro RA, Plentz RD. Inspiratory muscle training and quality of life in patients with heart failure: systematic review of randomized trials. Int J Cardiol. 2012;156(1):120-121. doi:10.1016/j.ijcard.2012.01.025	Systematic review.
Bonne G, Leturcq F, Ben Yaou R. Emery-Dreifuss Muscular Dystrophy. In: Adam MP, Ardinger HH, Pagon RA, et al., eds. GeneReviews®. Seattle (WA): University of Washington, Seattle; 1993.	Other conditions in addition to HF such as Emery-Dreifuss Muscular Dystrophy.
Padula CA, Yeaw E. Inspiratory muscle training: integrative review of use in conditions other than COPD. Res Theory Nurs Pract. 2007;21(2):98-118. doi:10.1891/088971807780852039	Review
Laoutaris ID, Dritsas A, Adamopoulos S, et al. Benefits of physical training on exercise capacity, inspiratory muscle function, and quality of life in patients with ventricular assist devices long-term postimplantation. Eur J Cardiovasc Prev Rehabil. 2011;18(1):33-40. doi:10.1097/HJR.0b013e32833c0320	Other conditions in addition to HF such as ventricular assist devices long-term post-implantation.
Rogers FJ. The muscle hypothesis: a model of chronic heart failure appropriate for osteopathic medicine. J Am Osteopath Assoc. 2001;101(10):576-583.	Non-experimental studies.
Samara AP, Lambrinou E. Home-based inspiratory muscle training in chronic heart failure: a systematic review. European journal of heart failure 2014; 16(null): 188-.	Systematic review.
Sbruzzi G, Dal Lago P, Ribeiro RA, Plentz RD. Efficacy of inspiratory muscle training in chronic heart failure patients. Int J Cardiol. 2012;161(2):119-120. doi:10.1016/j.ijcard.2012.06.035	Non-experimental studies.
NCT02794935 . The Effects of Inspiratory Muscle Training in Patients With Heart Failure and Obstructive Sleep Apnea Syndrome. https://clinicaltrials.gov/show/NCT02794935 2016	Other conditions in addition to HF such as obstructive sleep apnea syndrome.
Montemezzo D, Fregonezi GA, Pereira DA, Britto RR, Reid WD. Influence of inspiratory muscle weakness on inspiratory muscle training responses in chronic heart failure patients: a systematic review and meta-analysis. Arch Phys Med Rehabil. 2014;95(7):1398-1407. doi:10.1016/j.apmr.2014.02.022	Systematic Review and Meta-Analysis.
Meyer K. Exercise training in heart failure: recommendations based on current research. Med Sci Sports Exerc. 2001;33(4):525-531. doi:10.1097/00005768-200104000-00004	Non-experimental studies.
Arutyunov GP, Kolesnikova EA, Rylova AK, Rylova NV, Lobzeva VI. Respiratory muscle trainings in patients after myocardial infarction with concomitant heart failure. European respiratory society annual congress, barcelona, spain, september 18-22 2010	Other conditions in addition to HF such as interventions after myocardial infarction
Gayda M, Ribeiro PA, Juneau M, Nigam A. Comparison of Different Forms	Non-experimental studies.

of Exercise Training in Patients With Cardiac Disease: Where Does High-Intensity Interval Training Fit?. Can J Cardiol. 2016;32(4):485-494. doi:10.1016/j.cjca.2016.01.017	
Chen YM, Yin T. Inspiratory muscle training improves submaximal exercise capacity in patients with heart failure: a systematic review of randomized controlled trials. Int J Cardiol. 2012;158(2):294-296. doi:10.1016/j.ijcard.2012.04.126	Systematic review.
NCT03923153 . Effectiveness of Inspiratory Muscle Training in Patient With Stable Angina. https://clinicaltrials.gov/ct2/show/NCT03923153 2019	Other conditions in addition to HF such as stable angina.
Laoutaris ID, Adamopoulos S, Manginas A, Panagiotakos DB, Cokkinos DV, Dritsas A. Inspiratory work capacity is more severely depressed than inspiratory muscle strength in patients with heart failure: Novel applications for inspiratory muscle training. Int J Cardiol. 2016;221:622-626. doi:10.1016/j.ijcard.2016.07.102	Non-experimental studies.
Laoutaris ID, Dritsas A, Kariofyllis P, Manginas A. Benefits of inspiratory muscle training in patients with pulmonary hypertension: A pilot study [published online ahead of print, 2016 Aug 20]. Hellenic J Cardiol. 2016;S1109-9666(16)30155-5. doi:10.1016/j.hjc.2016.05.008	Other conditions in addition to HF such as pulmonary hypertension.
Smart NA. How do cardiorespiratory fitness improvements vary with physical training modality in heart failure patients? A quantitative guide. Exp Clin Cardiol. 2013;18(1):e21-e25.	Non-experimental studies.
NCT03186092 . Effects of Respiratory Muscle Training on Respiratory Muscle Strength, Functional Capacity and Quality of Life in Pulmonary Hypertension. https://clinicaltrials.gov/show/NCT03186092 2017	Other conditions in addition to HF such as pulmonary hypertension.
NCT02579200 . Inspiratory Muscle Training for Breathless Patients With Chronic Obstructive Pulmonary Disease and Heart Failure. https://clinicaltrials.gov/show/NCT02579200 2015	Other conditions in addition to HF such as chronic obstructive pulmonary disease.
Smart NA, Steele M. The effect of physical training on systemic proinflammatory cytokine expression in heart failure patients: a systematic review. Congest Heart Fail. 2011;17(3):110-114. doi:10.1111/j.1751-7133.2011.00217.x	Systematic review.
Laoutaris ID, Dritsas A, Adamopoulos S, Brown MD, Cokkinos DV. Effects of inspiratory muscle training in patients with chronic heart failure. J Am Coll Cardiol. 2008;52(23):1888-1889. doi:10.1016/j.jacc.2008.08.044	Non-experimental studies.
Arena R, Lavie CJ, Borghi-Silva A, et al. Exercise Training in Group 2 Pulmonary Hypertension: Which Intensity and What Modality. Prog Cardiovasc Dis. 2016;59(1):87-94. doi:10.1016/j.pcad.2015.11.005	Other conditions in addition to HF such as pulmonary hypertension.
NCT04173689 . Pulmonary Sarcoidosis With Exercise and Inspiratory Muscle Training. https://clinicaltrials.gov/show/NCT04173689 2019	Other conditions in addition to HF such as pulmonary sarcoidosis.
NCT02125760 . Respiratory Muscle Training in Subacute Stroke Patients. https://clinicaltrials.gov/show/NCT02125760 2014	Other conditions in addition to HF such as stroke.
Smetana GW, Conde MV. Preoperative pulmonary update. Clin Geriatr Med. 2008;24(4):607-vii. doi:10.1016/j.cger.2008.06.004	Other conditions in addition to HF such as preoperative pulmonary.
Reis MS, Arena R, Archiza B, de Toledo CF, Catai AM, Borghi-Silva A. Deep breathing heart rate variability is associated with inspiratory muscle weakness in chronic heart failure. Physiother Res Int. 2014;19(1):16-24. doi:10.1002/pri.1552	Non-experimental studies.
Laoutaris ID, Dritsas A, Brown MD, et al. Inspiratory muscle training in a patient with left ventricular assist device. Hellenic J Cardiol. 2006;47(4):238-241.	Other conditions in addition to HF such as left ventricular assist device.
Sandek A, von Haehling S, Anker SD. Muscle in heart disease: highlights from the European Society of Cardiology's Annual Meeting 2012. Int J Cardiol. 2012;161(3):126-129. doi:10.1016/j.ijcard.2012.10.014	Non-experimental studies.
Smart NA, Giallauria F, Dieberg G. Response to commentary "efficacy of inspiratory muscle training in chronic heart failure patients". Int J Cardiol. 2013;164(2):253-254. doi:10.1016/j.ijcard.2012.06.073	Non-experimental studies.
Souza Leão M, Mellaci Bergamascki L, Bertoni Xavier V, Boemo Jaenisch R, Stirbulov R, dos Santos Alves Vera L. Inspiratory muscle training in pulmonary hypertension: TREMMI protocol. Manual therapy, posturology & rehabilitation journal 2018; 16(null): 1-5.	Other conditions in addition to HF such as pulmonary hypertension.

doi:10.17784/mtprehabjournal.2018.16.569	
NCT03597646 . The Effect of Kinesio Taping on Pulmonary Function and Functional Capacity in Patients With Chronic Heart Failure. https://clinicaltrials.gov/show/NCT03597646 2018	Not used IMT.
NCT03491111 . The Outcome of Respiratory Muscle Training in Stroke Patients. https://clinicaltrials.gov/show/NCT03491111 2018	Other conditions in addition to HF such as stroke.
Laoutaris ID, Adamopoulos S, Manginas A, et al. Benefits of combined aerobic/resistance/inspiratory training in patients with chronic heart failure. A complete exercise model? A prospective randomised study. Int J Cardiol. 2013;167(5):1967-1972. doi:10.1016/j.ijcard.2012.05.019	Non-experimental studies.
RBR-42rmqy . Inspiratory muscle training in chronic obstructive pulmonary disease oxygen-dependent patients: a randomized controlled trial. http://www.who.int/trialsearch/Trial2.aspx?TrialID=RBR-42rmqy 2017	Other conditions in addition to HF such as chronic obstructive pulmonary disease.
Achttien RJ, Staal JB, van der Voort S, et al. Exercise-based cardiac rehabilitation in patients with chronic heart failure: a Dutch practice guideline. Neth Heart J. 2015;23(1):6-17. doi:10.1007/s12471-014-0612-2	Non-experimental studies.
RBR-3wss27 . Effects of Respiratory Muscle Training in Heart Disease and Sleep Apneia. http://www.who.int/trialsearch/Trial2.aspx?TrialID=RBR-3wss27 2018	Other conditions in addition to HF such as sleep apneia.
Kolesnikova E, Arutyunov G, Kostyukevich O, Rylova A. Respiratory muscles trainings are effective in lowering the pneumonia frequency in patients with heart failure and chronic obstructive pulmonary disease. European respiratory journal 2016; 48(null): null.	Other conditions in addition to HF such as chronic obstructive pulmonary disease.
Mahler DA, Barlow PB, Matthay RA. Chronic obstructive pulmonary disease. Clin Geriatr Med. 1986;2(2):285-312.	Other conditions in addition to HF such as chronic obstructive pulmonary disease.
NCT00815178 . Effects of Inspiratory Muscle Training on Type 2 Diabetes Mellitus Patients With Inspiratory Muscle Weakness. https://clinicaltrials.gov/show/NCT00815178 2008	Other conditions in addition to HF such as type 2 diabetes.
Kolesnikova E, Arutyunov G, Rylova A, Rylova N. Respiratory muscle trainings started in acute period of complicated myocardial infarction in patients with pulmonary hypertension. Circulation 2011; 124(21 SUPPL. 1): null.	Other conditions in addition to HF such as pulmonary hypertension.
Mandak JS, McConnell TR. Pulmonary manifestations of chronic heart failure. J Cardiopulm Rehabil. 1998;18(2):89-93. doi:10.1097/00008483-199803000-00001	Non-experimental studies.
Thunstrom U, Faager G. Effects of home-based inspiratory muscle training on symptoms in adults hospitalized for communityacquired pneumonia. European respiratory journal 2016; 48(null): null.	Other conditions in addition to HF such as pneumonia.
NCT02584205 . Does Inspiratory Muscle Training Improve Functional Capacity in Subjects With Obstructive Sleep Apnea?. https://clinicaltrials.gov/show/NCT02584205 2015	Other conditions in addition to HF such as obstructive sleep apnea.
Ben Gal T, Piepoli MF, Corrà U, et al. Exercise programs for LVAD supported patients: A snapshot from the ESC affiliated countries. Int J Cardiol. 2015;201:215-219. doi:10.1016/j.ijcard.2015.08.081	Non-experimental studies.
Neidenbach RC, Oberhoffer R, Nagdyman N, Seitz U, Ewert P, Kaemmerer H, Hager A. Inspiratory muscle training in children after fontan operation increases oxygen saturation. Cogent medicine 2017; 4(1): null.	Other conditions in addition to HF such as Fontan operation.
ACTRN12618001837213 . Breathing Muscle Training in Parkinson's Disease. http://www.who.int/trialsearch/Trial2.aspx?TrialID=ACTRN12618001837213 2018	Other conditions in addition to HF such as Parkinson's disease.
Martin J, Schwartzman K. Respiratory medicine and research at McGill University: a historical perspective. Can Respir J. 2015;22(1):18-19. doi:10.1155/2015/829760	Non-experimental studies.
RBR-8w44f6 . Clinical trial of the effect of breathing exercise on expiratory force and abdominal muscle in stroke patients. http://www.who.int/trialsearch/Trial2.aspx?TrialID=RBR-8w44f6 2019	Other conditions in addition to HF such as stroke.
NCT03726905 . Complex Exercises of the Respiratory Muscles Plus Aerobic Training vs. Aerobic Training in Patients With CHF. https://clinicaltrials.gov/show/NCT03726905 2018	Not used IMT.

Darnley GM, Gray AC, McClure SJ, et al. Effects of resistive breathing on exercise capacity and diaphragm function in patients with ischaemic heart disease. Eur J Heart Fail. 1999;1(3):297-300. doi:10.1016/s1388-9842(99)00027-6	Other conditions in addition to HF such as chronic coronary artery disease.
NCT01410279 . Inspiratory Muscle Training in Pulmonary Hypertension. https://clinicaltrials.gov/show/NCT01410279 2011	Other conditions in addition to HF such as pulmonary hypertension.
NCT01556139 . Effectiveness of Respiratory Muscle Training by Spirotiger in Chronic Patients. https://clinicaltrials.gov/show/NCT01556139 2012	Other conditions in addition to HF such as chronic obstructive pulmonary disease.
Walsh JT, Andrews R, Johnson P, Phillips L, Cowley AJ, Kinnear WJ. Inspiratory muscle endurance in patients with chronic heart failure. Heart. 1996;76(4):332-336. doi:10.1136/hrt.76.4.332	Not used IMT.
Cavalheri V, Burtin C, Formico VR, et al. Exercise training undertaken by people within 12 months of lung resection for non-small cell lung cancer. Cochrane Database Syst Rev. 2019;6(6):CD009955. Published 2019 Jun 17. doi:10.1002/14651858.CD009955.pub3	Other conditions in addition to HF such as lung resection.
Ambrosetti M, Doherty P, Faggiano P, et al. Characteristics of structured physical training currently provided in cardiac patients: insights from the Exercise Training in Cardiac Rehabilitation (ETCR) Italian survey. Monaldi Arch Chest Dis. 2017;87(1):778. Published 2017 May 18. doi:10.4081/monaldi.2017.778	Non-experimental studies.
Smart NA, Giallauria F, Dieberg G. Efficacy of inspiratory muscle training in chronic heart failure patients: a systematic review and meta-analysis. Int J Cardiol. 2013;167(4):1502-1507. doi:10.1016/j.ijcard.2012.04.029	Systematic Review and Meta-Analysis.
McConnell TR, Mandak JS, Sykes JS, Fesniak H, Dasgupta H. Exercise training for heart failure patients improves respiratory muscle endurance, exercise tolerance, breathlessness, and quality of life. J Cardiopulm Rehabil. 2003;23(1):10-16. doi:10.1097/00008483-200301000-00003	Not used IMT.
Ribeiro JP, Chiappa GR, Callegaro CC. The contribution of inspiratory muscles function to exercise limitation in heart failure: pathophysiological mechanisms. Rev Bras Fisioter. 2012;16(4):261-267. doi:10.1590/s1413-35552012005000034	Non-experimental studies.
JPRN-UMIN000029491 . Effects of inspiratory muscle training on exercise tolerance in hospitalized patients with chronic heart failure. http://www.who.int/trialsearch/Trial2.aspx?TrialID=JPRN-UMIN000029491 2017	Other conditions in addition to HF such as the patients are hospitalized.
Nava S, Fasano L. Inspiratory muscle training in difficult to wean patients: work it harder, make it better, do it faster, makes us stronger. Crit Care. 2011;15(2):153. doi:10.1186/cc10125	Not patients with HF.
Arena R, Pinkstaff S, Wheeler E, Peberdy MA, Guazzi M, Myers J. Neuromuscular electrical stimulation and inspiratory muscle training as potential adjunctive rehabilitation options for patients with heart failure. J Cardiopulm Rehabil Prev. 2010;30(4):209-223. doi:10.1097/HCR.0b013e3181c56b78	Review.
NCT02614001 . Inspiratory Muscle Training in Stroke Patients With Stable Congestive Heart Failure: a Prospective RCT. https://clinicaltrials.gov/show/NCT02614001 2015	Other conditions in addition to HF such as stroke.
Lin SJ, McElfresh J, Hall B, Bloom R, Farrell K. Inspiratory muscle training in patients with heart failure: a systematic review. Cardiopulm Phys Ther J. 2012;23(3):29-36.	Systematic review.
Aslan GK, Akıncı B, Yeldan I, Okumus G. A randomized controlled trial on inspiratory muscle training in pulmonary hypertension: Effects on respiratory functions, functional exercise capacity, physical activity, and quality of life [published online ahead of print, 2020 Mar 3]. Heart Lung. 2020;S0147-9563(20)30057-1. doi:10.1016/j.hrtlng.2020.01.014	Other conditions in addition to HF such as pulmonary hypertension.
Chen PC, Liaw MY, Wang LY, et al. Inspiratory muscle training in stroke patients with congestive heart failure: A CONSORT-compliant prospective randomized single-blind controlled trial [published correction appears in Medicine (Baltimore). 2017 Feb 17;96(7):e6212]. Medicine (Baltimore). 2016;95(37):e4856. doi:10.1097/MD.0000000000004856	Other conditions in addition to HF such as stroke.

Palau P, Domínguez E, López L, et al. Inspiratory Muscle Training and Functional Electrical Stimulation for Treatment of Heart Failure With Preserved Ejection Fraction: Rationale and Study Design of a Prospective Randomized Controlled Trial. Clin Cardiol. 2016;39(8):433-439. doi:10.1002/clc.22555	Non-experimental studies.

Abbreviations: HF, heart failure; IMT, inspiratory muscle training.
